# Genomic signatures of Lake Erie bacteria suggest interaction in the *Microcystis* phycosphere

**DOI:** 10.1371/journal.pone.0257017

**Published:** 2021-09-22

**Authors:** Alexa K. Hoke, Guadalupe Reynoso, Morgan R. Smith, Malia I. Gardner, Dominique J. Lockwood, Naomi E. Gilbert, Steven W. Wilhelm, Isabella R. Becker, Grant J. Brennan, Katherine E. Crider, Shannon R. Farnan, Victoria Mendoza, Alison C. Poole, Zachary P. Zimmerman, Lucy K. Utz, Louie L. Wurch, Morgan M. Steffen

**Affiliations:** 1 James Madison University, Harrisonburg, VA, United States of America; 2 Virginia Tech, Blacksburg, VA, United States of America; 3 Texas A&M University, College Station, TX, United States of America; 4 University of Tennessee, Knoxville, TN, United States of America; INRA/Sorbonne University, FRANCE

## Abstract

Microbial interactions in harmful algal bloom (HAB) communities have been examined in marine systems, but are poorly studied in fresh waters. To investigate HAB-microbe interactions, we isolated bacteria with close associations to bloom-forming cyanobacteria, *Microcystis* spp., during a 2017 bloom in the western basin of Lake Erie. The genomes of five isolates (*Exiguobacterium* sp. JMULE1, *Enterobacter* sp. JMULE2, *Deinococcus* sp. JMULE3, *Paenibacillus* sp. JMULE4, and *Acidovorax* sp. JMULE5.) were sequenced on a PacBio Sequel system. These genomes ranged in size from 3.1 Mbp (*Exiguobacterium* sp. JMULE1) to 5.7 Mbp (*Enterobacter* sp. JMULE2). The genomes were analyzed for genes relating to critical metabolic functions, including nitrogen reduction and carbon utilization. All five of the sequenced genomes contained genes that could be used in potential signaling and nutrient exchange between the bacteria and cyanobacteria such as *Microcystis*. Gene expression signatures of algal-derived carbon utilization for two isolates were identified in *Microcystis* blooms in Lake Erie and Lake Tai (*Taihu*) at low levels, suggesting these organisms are active and may have a functional role during *Microcystis* blooms in aggregates, but were largely missing from whole water samples. These findings build on the growing evidence that the bacterial microbiome associated with bloom-forming algae have the functional potential to contribute to nutrient exchange within bloom communities and interact with important bloom formers like *Microcystis*.

## Introduction

Cyanobacterial harmful algal blooms (cHABs) occur annually in both freshwater and marine systems. These blooms have the potential to be disruptive to aquatic ecosystems due to both the scale of accumulated biomass and the release of secondary metabolites that have metabolic consequences for other organisms [1, 2]. *Microcystis* is a pervasive genus of cyanobacteria that forms blooms on every continent except Antarctica [2]. Some species of *Microcystis* produce microcystins, potent hepatotoxins that can limit access to potable water [3, 4]. The threats to ecosystem services and public health posed by cHABs have resulted in numerous and diverse mitigation and management strategies ranging from simple aeration of small freshwater systems [5] to application of chemicals [6, 7] or barley straw [8]. In the last decade, considerable advancements have been made in the application of biotic solutions that inhibit cHABs, including those based on microorganisms, although these technologies have yet to be successfully validated beyond laboratory-scale, albeit environmentally relevant, studies [9]. Several bacteria have been identified that are capable of degrading microcystins produced by cyanobacteria or are algicidal [10–12]. Previous work suggests such antagonistic interactions may occur in the *Microcystis* phycosphere [13, 14], a microenvironment that surrounds phytoplankton cells analogous to the rhizosphere in plant roots [15]. However, bacterial-phytoplankton interactions are not exclusively antagonistic, as evidence from marine phytoplankton studies suggests mutualistic relationships also exist [16]. The phycosphere provides a nutrient-rich environment for heterotrophic bacteria due to the release of organic molecules by the phytoplankton, including dissolved organic carbon [17]. In the early phases of cell growth, phytoplankton release lower molecular weight molecules, such as amino acids and carbohydrates, while higher molecular weight molecules, such as polysaccharides, nucleic acids, and proteins, can be released into the phycosphere during lysis [17]. The release of molecules by phytoplankton can attract heterotrophic bacteria to the phycosphere, ultimately leading to a potential exchange of nutrients between the bacteria and phytoplankton [16, 17].

The physiology of *Microcystis* spp. makes them well-suited for nutrient exchange with heterotrophic bacterial partners. *Microcystis* spp. are colonial cyanobacteria surrounded by an exopolysaccharide layer with which bacteria are tightly coupled [18–20]. Previously, multiple strains of bacteria have been found to impact the formation of *Microcystis* colonies and exopolysaccharide production [21]. Furthermore, the species of bacteria associated with the *Microcystis* phycosphere differ based on whether they are particle associated (> 10 μm) or free-living [22]. While we know that heterotrophic bacteria are closely associated with *Microcystis* colonies [23], the potential mechanisms of exchange between the partners in the freshwater cHAB phycosphere have yet to be characterized. In the current study we provide genomic data that support the previously proposed interaction by which heterotrophic bacteria (heterobionts) utilize the carbon released by *Microcystis*, while *Microcystis* may benefit from nutrient or vitamin products released by the heterotrophic bacteria [23].

Here, we report on the genomic content of five bacterial strains isolated from *Microcystis* aggregates in western Lake Erie in August 2017, focusing on the genetic potential for interaction with *Microcystis*. Access to the genomic information of *Microcystis*-associated heterotrophic bacteria has provided new insight into the potential microbial interactions and metabolic pathways that occur within *Microcystis* blooms, specifically that nutrient exchange may occur in the *Microcystis* phycosphere. To demonstrate the ecological relevance of these strains, we surveyed available metatranscriptomic data from *Microcystis* spp. blooms in North America and China. These observations show the potential for bidirectional, mutualistic interactions in the *Microcystis* phycosphere which could serve as a future target for cHAB mitigation.

## Results and discussion

### Sample collection and environmental conditions

Surface samples were collected from four stations in the Western Basin of Lake Erie in August 2017 (WE02, WE04, WE13, and MB18) using 20 μm and 80 μm mesh plankton nets. These pore sizes were chosen to exclude free-living bacteria and enrich for those bacteria that are associated with *Microcystis* aggregates. Previous studies have characterized *Microcystis*-associated bacteria in size fractions ranging from ≥ 3 μm up to 100 μm. Environmental conditions at the time of sample collection were reported by Boedecker et al. [24].

#### Isolate characteristics

Five isolates were targeted for full genome sequencing from a library of over 100 individual isolates generated from the Lake Erie bloom samples. These isolates were selected based on their N-utilization and pigment production capabilities. The five isolates selected to be sequenced were identified at the genus level *via* 16S rRNA and *rpoB* gene sequences from genomic data as an *Exiguobacterium* sp. (JMULE1), an *Enterobacter* sp. (JMULE2), a *Deinococcus* sp. (JMULE3), a *Paenibacillus* sp. (JMULE4), and an *Acidovorax* sp. (JMULE5) (**S1-S5 Figs in S1 File)**. *Exiguobacterium* sp. JMULE1 is a gram-positive, rod-shaped, motile bacterium that produces orange pigmented colonies. Members of this genus have previously been shown to impact colony formation in individual strains of *Microcystis*, both positively [25] and negatively [26], depending on the strains tested. *Enterobacter* sp. JMULE2 is a gram-negative, rod-shaped bacterium from the class Gammaproteobacteria. Multiple *Enterobacter* strains have been found to have microcystin degradation capabilities and induce cell aggregation by *Microcystis* [21, 27, 28]. *Deinococcus* sp. JMULE3 is a gram-positive rod-shaped bacterium belonging to the class Deinococci that produces pink-orange pigmented colonies; members of the Deinococcus-Thermus phylum have been previously identified in *Microcystis* metagenomes [13, 29]. *Paenibacillus* sp. JMULE4 is a gram-negative, rod-shaped bacterium in the class Bacilli that produces endospores, and members of this genus have been previously identified in cyanobacterial bloom communities [30]. Isolates of *Paenibacillus* are commonly applied as algal bioflocculants, as they induce algal cell aggregation [31, 32]. *Acidovorax* sp. JMULE5 is a gram-negative, rod-shaped bacterium belonging to the class Betaproteobacteria. Several strains of this genus have been isolated from samples of *Microcystis*, both in culture [33] and from the environment [34].

The microbiome of freshwater lakes and rivers is often dominated by members of the phylum Actinobacteria. While none of the bacteria isolated for this study were members of the Actinobacteria phylum, this is consistent with recent work demonstrating that this phylum is significantly depleted in populations closely associated with the *Microcystis* phycosphere [35]. In fact, members of the phyla Proteobacteria and Firmicutes are enriched in *Microcystis* aggregate samples compared to free water bloom samples in several studies [22, 25, 35, 36]. It has been hypothesized that Actinobacteria likely do not rely on *Microcystis*-derived carbon due to actinorhodopsin activity [35, 37]. Furthermore, one benefit to phycosphere bacteria may be protection from predation by zooplankton, to which ultramicrobacterial Actinobacteria are not as vulnerable [38, 39].

### Sequencing output, assembly, and annotation

The number of raw reads ranged from 197,286 (*Deinococcus* sp. JMULE3) to 455,299 reads (*Enterobacter* sp. JMULE2) (**S1 Table in S1 File**). Read correction done within the PacBio *de novo* assembly pipeline resulted in 27,959 (*Deinococcus* sp. JMULE3) to 69,289 reads (*Enterobacter* sp. JMULE2) (**S1 Table in S1 File**). Genome completeness was assessed with the PATRIC Genome Assembly tool and ranged from 98.2% (*Paenibacillus* sp. JMULE4) to 100% (*Enterobacter* sp. JMULE2 and *Acidovorax* sp. JMULE5) (**Table 1**).

**Table 1:** Characteristics of genome assemblies obtained from the PacBio *de novo* assembly pipeline in the CLC Genome Finishing Module.

Isolate	# of Contigs	N50 (Mbp)	GC %	Total Length (Mbp)	Coding Sequences	Genome Completeness
*Exiguobacterium* sp. JMULE1	2	3.11	47.13	3.15	3,289	99.5%
*Enterobacter* sp. JMULE2	20	0.61	54.79	5.74	5,736	100%
*Deinococcus* sp. JMULE3	5	3.28	69.75	4.22	4,208	99.5%
*Paenibacillus* sp. JMULE4	19	0.39	49.95	5.40	5,888	98.2%
*Acidovorax* sp. JMULE5	1	5.45	64.48	5.45	5,101	100%

*Acidovorax* sp. JMULE5 was the only isolate for which a single contig was obtained (**Table 1**). Its closest sequenced relative, *Acidovorax* sp. KKS102 was originally isolated from soil and has been shown to degrade polychlorobiphenyl (PCB) (**Table 2** [40]). Recently, a strain of *Acidovorax* was isolated from a *Microcystis* bloom in Korea, but genomic information is not currently available for this isolate [33]. At 5,742,593 bp, the genome of *Enterobacter* sp. JMULE2 is comparable to the most closely related isolate based on *rpoB* identity, *Enterobacter asburiae* sp. L1 (~5.4 Mbp; **Table 2** [41]). Based on *rpoB* identity and the two-way average nucleotide identity between the two genomes [42], it is unlikely that *E*. *asburiae* sp. L1 and the JMULE2 isolate are the same species (**Table 2**) [43]. However, *Exiguobacterium* sp. JMULE1 is likely the same species as its most closely related sequence isolate, *Exiguobacterium* sp. MH3, with a two-way ANI score > 95% (**Table 2**) [43]. *Exiguobacterium* sp. MH3 was isolated from the rhizosphere of duckweed (*Lemna minor*) and has both growth promoting and stress alleviating effects on its freshwater eukaryotic host [44, 45]. *Paenibacillus napthalovorans* sp. 32-OY is likely also the same species as *Paenibacillus* JMULE4, with an ANI score > 95% (**Table 2**). *P*. *napthalovorans* sp. 32-OY was originally isolated from soil and can metabolize dibenzothiophene, an organosulfur compound [46, 47]. The ability to degrade high molecular weight compounds is a signature of bacteria associated with *Microcystis* aggregates and may indicate an important role in the transformation of algal-derived organic compounds in bloom communities [22, 48].

**Table 2:** Closest sequenced relatives of each isolate based on *rpoB* identity and ANI score.

Lake Erie Isolate	Closest sequenced relative	Genome Size (Mbp)	GC %	Genes	*rpoB* % Identity	Two-Way ANI Score (%)	Citation
*Exiguobacterium* sp. JMULE1	*Exiguobacterium* sp. Strain MH3	3.16	47.2	3,273	99.8	98.2	Tang et al., 2013
*Enterobacter* sp. JMULE2	*Enterobacter asburiae* sp. L1	4.56	56.1	4,426	98.5	90.0	Lau *et al*. 2014
*Deinococcus* sp. JMULE3	*Deinococcus soli* N5	3.24	70.2	3,146	97.0	92.4	Joo et al., 2015
*Paenibacillus* sp. JMULE4	*Paenibacillus napthalenovorans*32O-Y	5.20	49.7	5,103	99.5	99.3	Butler et al., 2016)
*Acidovorax* sp. JMULE5	*Acidovorax* sp. KKS102	5.20	64.9	4,883	92.5	87.3	Ohtsubo et al., 2012

### Functional annotation

**Fig 1:**
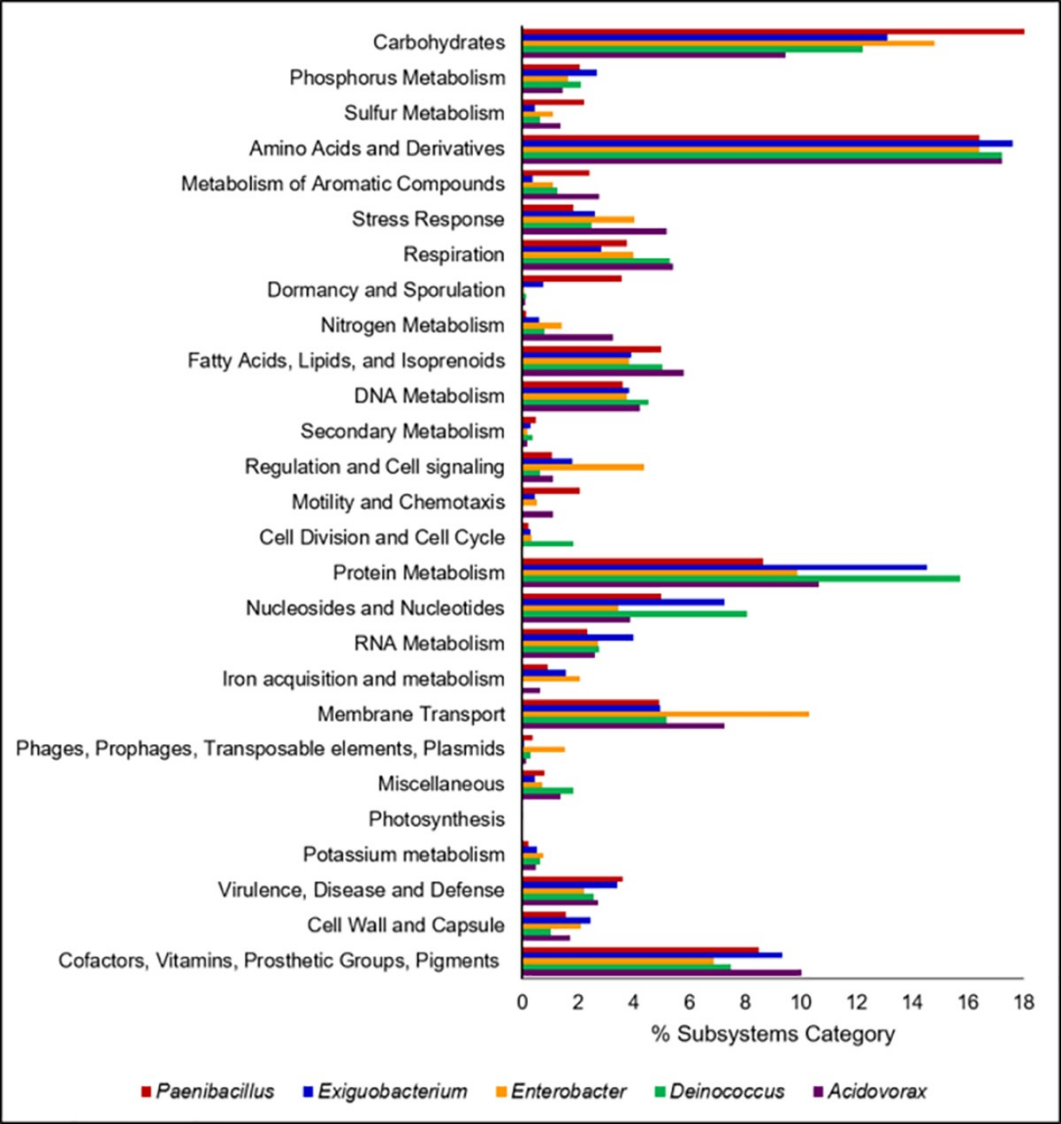
Percent of annotated genes in each Subsystems (SEED) category. Subsystem coverage for each of the isolates was 30% for *Exiguobacterium* sp. JMULE1, 33% for *Enterobacter* sp. JMULE2, 23% for *Deinococcus* sp. JMULE3, 26% for *Paenibacillus* sp. JMULE4, and 31% for *Acidovorax* sp. JMULE5.

To better understand the dominant metabolic pathways encoded by each Lake Erie isolate, the protein families of each genome were annotated using the Subsystems (SEED) approach [49, 50]. Overall, the five genomes contain the highest number of subsystems related to the Carbohydrates, Amino Acids and Derivatives, and Protein Metabolism categories (**Fig 1**). The genome of *Paenibacillus* sp. JMULE4 contained the greatest number of pathways related to Carbohydrates, while *Exiguobacterium* sp. JMULE1 and *Deinococcus* sp. JMULE3 contained a proportionately greater number of pathways related to Protein Metabolism (**Fig 1**). As isolates of *Paenibacillus* have been shown to be capable of degrading complex carbohydrates, this may be indicative of similar capabilities for the Lake Erie isolate of this genus [51, 52] and bacteria associated with *Microcystis* aggregates [22, 36]. The *Paenibacillus* sp. JMULE4 genome also contained the most genes related to Dormancy and Sporulation, and this is the only isolate of the five observed to produce endospores (**Fig 1**).

### Nitrogen utilization

Bacterial heterobionts are thought to be a source of nitrogen (N) to algae in the phycosphere [35, 36, 53]. In many ways this is self-evident, as respiration of biological materials from phytoplankton results in the loss of C (as CO_2_) and residual, excess N [54, 55]. The presence of several different N-transformation genes in the bacterial genomes we examined suggests that these bacteria have the capability to act as an external source of ammonium for *Microcystis*. All the genomes except *Exiguobacterium* sp. JMULE1 contain genes for the reduction of nitrate and nitrite to ammonium (**Table 3; S2-S6 Tables in S1 File**). Enrichment for this function has been previously identified in metagenomes generated from *Microcystis* aggregates in Lake Erie compared to whole water samples [35]. The denitrifying reductase gene clusters in the *Acidovorax* sp. JMULE5 genome includes genes for nitric and nitrous oxide reductase as well as cyanate hydrolysis (**Table 3; S2 Table in S1 File**). Cyanate is a by-product of the urea cycle and produces bicarbonate and ammonium ions upon hydrolysis via the enzyme cyanase (*cynS*) [56]. *Microcystis* populations have been shown to upregulate transcription of *cynS* in response to urea additions [57], indicating *Microcystis* has the genetic capability to use cyanate derived from associated bacterial populations in systems such as Lake Erie during periods of N limitation.

**Table 3:** Nitrogen genes called by RAST and PGAP and their roles in the bacterial isolates.

Gene	Role	Isolate(s)
*nirD*	Nitrate reductase small subunit	JMULE2, JMULE3, JMULE4, JMULE5
*nirB*	Nitrate reductase large subunit	JMULE2, JMULE3, JMULE4, JMULE5
*narG*	Respiratory nitrate reductase alpha chain	JMULE2, JMULE5
*narH*	Respiratory nitrate reductase beta chain	JMULE2, JMULE5
*narI*	Respiratory nitrate reductase gamma chain	JMULE2, JMULE5
*narJ*	Respiratory nitrate reductase delta chain	JMULE2
*norR*	Anaerobic nitric oxide reductase transcription regulator	JMULE2, JMULE5
*nsrR*	Nitrite-sensitive transcriptional repressor	JMULE1, JMULE2
*gltB*	Glutamate synthase large chain	JMULE1, JMULE3 JMULE5
*gltD*	Glutamate synthase small chain	JMULE1, JMULE3 JMULE5
*glnN*	Glutamine synthetase type III	JMULE3
*cynS*	Cyanate hydratase	JMULE5
*cynR*	Cyn operon transcriptional activator	JMULE5
*nosF nosR*, *nosY*	Nitrous oxide reductase maturation protein	JMULE5
*norB*	Nitric-oxide reductase subunit B	JMULE5

While previous work has identified diazotrophic bacterial constituents of *Microcystis* blooms and culture consortia, none of the five Lake Erie isolates have the genetic capacity to produce nitrogenases [20, 23, 58]. Members of the genus *Paenibacillus* have the ability to fix N_2_ [59, 60], however, the JMULE4 isolate only encodes a NifU-like protein, which is a nonessential protein for nitrogen fixation in organisms such as *Dolichospermum* (*Anabaena*) [61]. The role of these isolates in potentially providing reduced N to *Microcystis* likely comes from the breakdown other exogenous N sources, such as urea and nitrate.

Urease is an enzyme that catalyzes the hydrolysis of urea to ammonia and carbon dioxide (CO_2_) [62]. In addition to serving as an N source for freshwater cyanobacteria, including *Microcystis*, the CO_2_ released during urea hydrolysis also can act as a carbon source for *Microcystis* during periods of high biomass [63, 64]. As the pH increases during bloom events, it becomes increasingly difficult for additional CO_2_ to dissolve in the water. CO_2_ availability can be impacted by static conditions (no aeration), which can cause *Microcystis* to stop growing [65]. The genomes of *Enterobacter*, *Paenibacillus*, and *Acidovorax* spp. contain genes encoding the alpha (*ureC*), beta (*ureB*), and gamma (*ureA*) subunits of the urease enzyme complex. The *Deinococcus* sp. JMULE3 genome contains genes for the alpha (*ureC*) and gamma (*ureA*) urease subunits. The *Deinococcus* sp. JMULE3 and *Acidovorax* sp. JMULE5 genomes contain all of the urease accessory genes (*ureEFGD*), while the *Enterobacter* sp. JMULE2 and *Paenibacillus* sp. JMULE4 genomes encode a subset of these accessory genes (*ureEFD* and *ureFGD* respectively). *Enterobacter* sp. JMULE2, *Deinococcus* sp. JMULE3, and *Acidovorax* sp. JMULE5 were all confirmed to be ureolytic by inducing a color change on urea slants and can grow with urea as their sole N source. Urease is a metalloenzyme that binds to and requires nickel to function [66, 67]. The genomes of *Enterobacter* sp. JMULE2 and *Acidovorax* sp. JMULE5 contain the nickel-binding accessory genes *ureJ* and *hupE*. Genes for nickel incorporation proteins (*hypAB*) were identified in the *Enterobacter* sp. JMULE2 and *Deinococcus* sp. JMULE3 genomes. Due to its potential as a source of CO_2_, the breakdown of urea by heterotrophic bacteria in the phycosphere could act as a dual source of both carbon and N for *Microcystis*, as has been shown in marine systems [68].

### Carbon utilization

The monosaccharide composition of *Microcystis* extracellular polysaccharides (EPS) has been extensively characterized, and multiple species of bacteria can use components of the *Microcystis* EPS as a sole carbon source [69–71]. The genomes of *Paenibacillus* sp. JMULE4 and *Enterobacter* sp. JMULE2 contain genes for xylose utilization, a key monosaccharide in the EPS of *Microcystis* [69, 70]. In both freshwater and marine phycosphere communities, xylose is a known low molecular weight (LMW) carbohydrate source for associated bacteria [72–74]. *Paenibacillus* sp. JMULE4 genome contains genes for xylose isomerase, transporters, and binding components, while *Enterobacter* sp. JMULE2 has genes encoding XylFGHR proteins that allow for transcriptional regulation, xylose transport, and ATP binding (**S2-S6 Tables in S1 File**). All five of the isolates’ genomes also contain genes for mannose utilization (**S2-S6 Tables in S1 File**), another component of the *Microcystis* EPS [69, 70]. All of the isolates have the genetic capacity to produce mannose-6-phosphate (M6P) isomerase (**S2-S6 Tables in S1 File**), which converts M6P to fructose-6-phosphate (F6P), an intermediate of glycolysis.

The genomes of *Deinococcus* sp. JMULE3, *Paenibacillus* sp. JMULE4, and *Acidovorax* sp. JMULE5 contain the *glcD* gene for glycolate dehydrogenase, indicating they likely have the ability to use algal-derived glycolate as a carbon source (**S2-S6 Tables in S1 File**). Glycolate is an organic carbon source produced from the oxygenase activity of RuBisCO during photorespiration by phytoplankton, with rates of excretion dependent upon the form of N available [73, 75]. The potential utilization of glycolate by heterotrophic bacteria is indicated by the presence of the *glcD* gene, which encodes glycolate oxidase; this gene is now considered a biomarker for the ability to consume algal-derived carbon [76, 77]. Bacterial utilization of glycolate released by phytoplankton has been examined in both marine systems and lakes [77, 78]. To determine whether these organisms actively attempt to access glycolate pools during bloom events, we examined the genetic potential of bloom communities to use this C source in metagenomes from Lake Greenfield (Iowa, USA) and the active transcription of *glcD* in a set of transcriptomes from Lake Erie (North America) and Lake Tai (*Taihu*) and (**Table 4**). Signatures of *Paenibacillus* or *Deinococcus glcD* expression are largely non-existent (**Table 4**), indicating they were not actively using (or capable of using) glycolate during the bloom events sampled. While overall few reads recruited to *glcD* from the bloom metatranscriptomes, the greatest number of reads recruited to the *Acidovorax* JMULE5 *glcD* gene, indicating that there is some active transcription of this gene during bloom events by this species and other members of the Comamonadaceae during bloom events in Taihu and Lake Erie (**Table 4**). The increased recruitment of reads from the Greenfield metagenomes is likely a function of samples being DNA rather than RNA, indicating the potential of these organisms to use glycolate, rather than active transcription. The low number of reads which recruited to the *glcD* sequences in these bloom libraries is likely a function of the sample collection, as these were all whole water samples rather than *Microcystis*-aggregates. Furthermore, little is known about seasonality of bacterial interactions in the phycosphere. There may be a specific bloom stage during which phycosphere bacteria may actively consume algal-derived carbon such as glycolate. These organisms are members of phyla that are significantly reduced in whole water samples compared to aggregates [14, 35], where Actinobacteria are universally dominant in freshwater systems [14, 79, 80]. Unfortunately, few if any datasets exist that measure gene expression specific to bacteria within *Microcystis* aggregates, although several recent studies have reconstructed bacterial functional potential within aggregates using metagenomics [22, 25, 35, 36]. Furthermore, it is likely that the type of interactions between *Microcystis* and its associated bacteria may vary between synergistic or mutualistic and antagonistic depending on the stage of bloom development [74]. Taken together, the content of these isolates’ genomes likely indicates carbon exchange in the *Microcystis* phycosphere.

**Table 4:** Metatranscriptome and metagenome reads recruited to the *glcD* gene of *Acidovorax* JMULE5, *Deinococcus* JMULE3, and *Paenibacillus* JMULE4. Libraries from Lake Erie metatranscriptomes (Steffen et al., 2017; Stough et al., 2019), Taihu metatranscriptomes (Stough et al., 2019), and Greenfield metagenomes were recruited to each *glcD* gene.

Lake	*Acidovorax* (Comamonadaceae)	*Deinococcus* (Denococcaceae)	*Paenibacillus* (Paenibacillaceae)
Taihu	12 (128)	0 (0)	0 (0)
Erie	38 (252)	0 (0)	0 (0)
Greenfield	3,205 (21,536)	0 (0)	4 (0)

### Iron utilization

Iron deprivation affects phytopigment production and photosynthetic efficiency of *Microcystis* spp. [81]. *Enterobacter* sp. JMULE2, *Paenibacillus* sp. JMULE4, and *Deinococcus* sp. JMULE3 genomes all contain genes encoding various siderophore transporters, biosynthetic pathways, and utilization proteins (**S3 Table in S1 File**).

The *Enterobacter* sp. JMULE2 genome contains genes encoding FepBCDEG proteins for the transport of ferric enterobactin (**S3 Table in S1 File**). Genes encoding the enterobactin biosynthesis pathway proteins EntBSH are also present (**S3 Table in S1 File**). Enterobactin siderophores are characteristic of the Enterobacteriaceae family and are amongst the strongest siderophores with a high affinity for iron [82]. The genomes of 115 *Microcystis aeruginosa* isolates in Genbank do not contain a gene encoding enterobactin esterase (*fes*), the enzyme necessary to remove iron from the enterobactin siderophore. If *Microcystis* spp. cannot use enterobactin, it is possible that iron scavenging by phycosphere bacteria could be competitive with their cyanobacterial host during specific phases of bloom development [74]. The *Enterobacter* sp. JMULE2 genome also contains *iucA-D* genes necessary for aerobactin synthesis (**S3 Table in S1 File**). Aerobactin siderophores do not have as strong an affinity for iron as enterobactins, but have an advantage for bacterial growth in iron-limited conditions [83]. Members of the bloom-forming genus *Dolichospermum* (formerly *Anabaena*) can use ferric aerobactin in culture, although it is not considered a robust iron donor for cyanobacteria [84].

The genome of *Deinococcus* sp. JMULE3 contains genes for isochorismate synthase (**S3 Table in S1 File**), a precursor of siderophores including enterobactin [85]. Isochorismate synthase is necessary for the synthesis of salicylic acid for plant defense [86]. The *Paenibacillus* sp. JMULE4 genome contains genes related to bacillibactin and anthrachelin siderophores (**S3 Table in S1 File**). The genome also contains genes for Feu A-C proteins for Fe-bacillibactin transport [87]. Bacillibactins are catechol-based siderophores that are structurally like enterobactin siderophores which are produced by different members of the *Bacillus* genus, including *Bacillus anthracis* [88]. These siderophores have also been described in a *Paenibacillus* honeybee pathogen [89]. *Paenibacillus* sp. JMULE4 genome contains genes for anthrachelin uptake transporters (**S3 Table in S1 File**).

The Ton and Tol transport systems are used to transport ferric-siderophore complexes and vitamin B_12_ across the cell membrane [90]. The genomes of *Acidovorax* sp. JMULE5, *Deinococcus* sp. JMULE3, and *Enterobacter* sp. JMULE2 contain genes involved in the Ton and Tol transport systems. All three organisms also have genes for TonB-dependent receptors (**S3 Table in S1 File**). *Enterobacter* sp. JMULE2 and *Acidovorax* sp. JMULE5 have genes for the TolA protein (**S3 Table in S1 File**). The *Enterobacter* sp. JMULE2 genome contains the gene encoding aerobactin siderophore receptor, *iutA* (**S3 Table in S1 File**).

### Production of auxins

**Fig 2:**
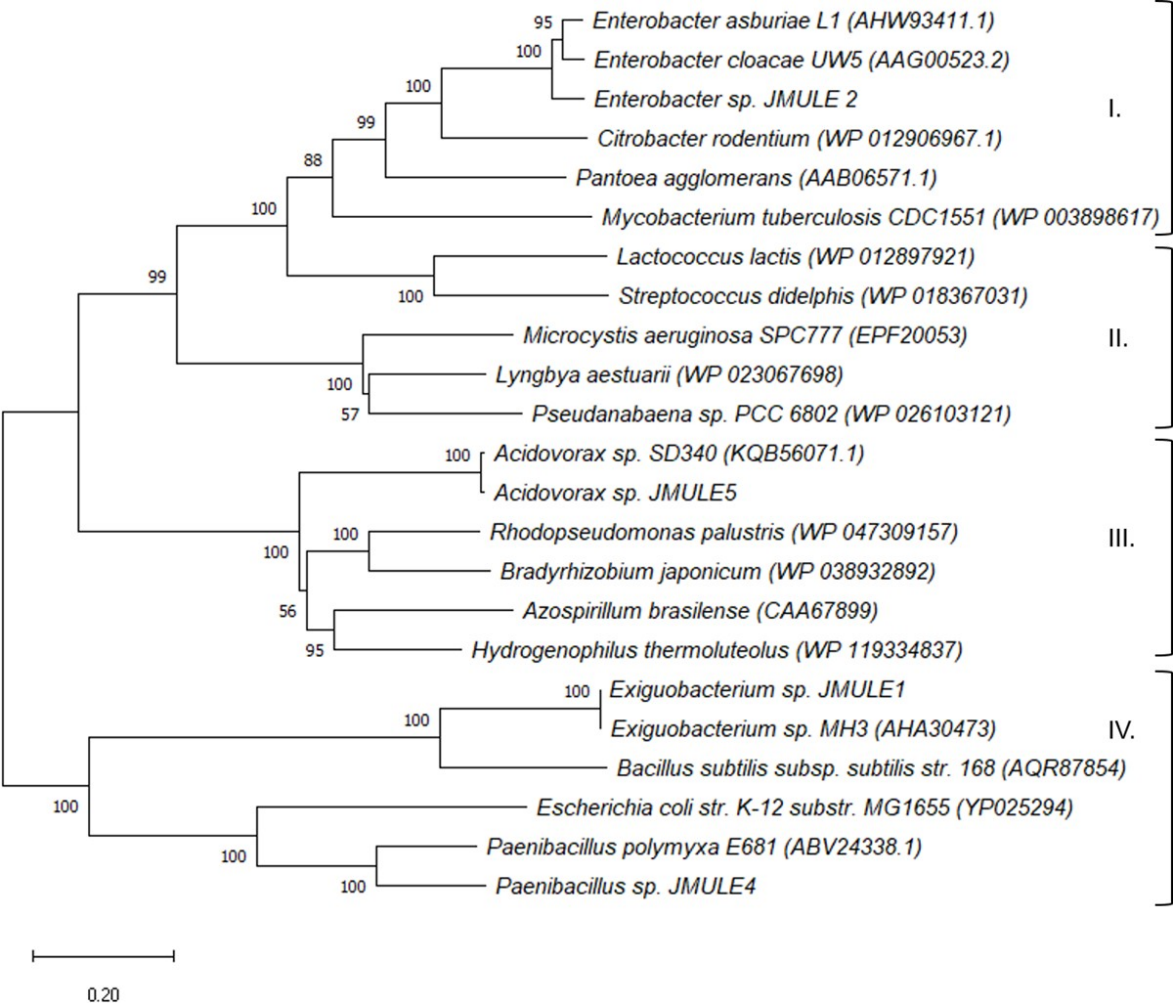
Phylogenetic tree of bacterial homologues of indolepyruvate decarboxylase (IpdC). Clusters are based on similarity to sequences of known function, specifically indolepyruvate decarboxylase (Group I), α-keto decarboxylase (Group II), acetolactate synthase (Group III), and phenylpyruvate decarboxylase (Group IV). Sequence alignment (527 amino acids) was performed using T-coffee (Notredame *et al*., 2000; Di Tomasso *et al*., 2011) and the Neighborhood-Joining phylogenetic tree was generated in Mega X (Kumar *et al*., 2018) with a bootstrap test of phylogeny (1000).

Each of the five sequenced bacterial genomes encode genes for the biosynthesis of tryptophan, and four encode homologues of *ipdC*, the gene which encodes indole-3-pyruvate decarboxylase (**S2-S5 Tables in S1 File; Fig 2**). Sequences of *ipdC* fall into four clusters based on similarity to sequences of known function; *Enterobacter* sp. JMULE2 belongs to cluster I, encoding indolepyruvate decarboxylase, along with other members of the *Enterobacter* genus and close relatives such as *Citrobacter* (**Fig 2**). *Acidovorax* sp. JMULE5 falls in cluster II, whose members encode an α-keto decarboxylase, while *Exiguobacterium* sp. JMULE1 and *Paenibacillus* sp. JMULE 4 belong to cluster IV, which encodes a phenylpyruvate decarboxylase (**Fig 2**). IpdC and its homologues catalyze the second reaction in the indole-3-pyruvic acid (IPA) IAA synthesis pathway, converting IPA to indole-3-acetaldehyde (IAAld) (**Fig 2**). When supplemented with 5 mM tryptophan, all five isolates were confirmed to produce IAA via colorimetric assay, with a range of 3.3 μM *(Exiguobacterium* sp. JMULE1) to 47.3 μM (*Acidovorax* sp. JMULE5) after a 24-hour incubation (**S6 Table in S1 File**). The closest relative of JMULE1, *Exiguobacterium* sp. MH3, produces auxins that are hypothesized to play a role in growth-promoting activity in its aquatic plant host [44]. Production of auxins by native bacteria increases cell density of freshwater eukaryotic microalgae [91]. Tryptophan is an important precursor for indole-3-acetic acid (IAA), the main auxin that occurs in plants. Amin et al. (2015) found that a bacterial consortium promoted diatom cell division due to the secretion of an auxin synthesized from diatom-derived tryptophan. *Microcystis aeruginosa* NIES 843 has the genetic capability to produce tryptophan and could therefore serve in a similar role [16, 92]. The production of auxins like IAA by these isolates suggests a possible important growth-promoting effect of the bacteria on *Microcystis* and other freshwater cHAB formers.

### Quorum sensing and signaling

The *Enterobacter* sp. JMULE2 genome contains genes for AI-2 transport and processing. Quorum sensing, or cell-to-cell communication in bacteria, relies on the production of signaling molecules known as autoinducers [93]. Autoinducer 2 (AI-2) is an autoinducer produced by many different bacterial species. This *lsrACDBFGE* operon has also been described in *Salmonella enterica* and *Escherichia coli* [94, 95], both members of the Enterobacteriaceae family with *Enterobacter*. In these organisms, the *lsrACDB* genes encode AI-2 transporter components while the rest of the genes in the operon are needed from processing AI-2 once it is internalized [95]. Its closest relative (based on *rpoB* identity), *E*. *asburiae* sp. L1 (**Table 3**) is known to produce an array of quorum sensing molecules, including AHLs [41].

### Vitamin production

Many algae, including some cyanobacteria [96–98], require vitamin B_12_ for growth yet are unable to produce it and must rely on exogenous B_12_ [97]. *Microcystis* requires vitamin B_12_ for the methionine biosynthesis pathway. This pathway requires B_12_ as a cofactor for a type-II MetH enzyme [48, 97]. Microalgae can obtain vitamin B_12_ directly from bacterial interactions [97]. *Paenibacillus* sp. JMULE4, *Acidovorax* sp. JMULE5, and *Deinococcus* sp. JMULE3 genomes all contain genes for cobalamin (vitamin B_12_) synthesis (MTR, *cobY*, *cobU*, *cobQ*, *bluB*) (**S1-S5 Tables in S1 File**). Vitamin B_7_ (biotin) is a cofactor that is essential for carboxylase enzymes, including acetyl coenzyme A (CoA) carboxylase which is used in the production of fatty acids. As with vitamin B_12_, cultures of *Microcystis* are supplemented with B_7_ in the growth medium. All five of the bacterial genomes contain genes for biotin biosynthesis (**S1-S5 Tables in S1 File**). In marine systems, 22% of HAB forming organisms are vitamin B_1_ (thiamine) auxotrophs [99]. The *Enterobacter* sp. JMULE2, *Paenibacillus* sp. JMULE4, and *Acidovorax* sp. JMULE5 genomes contain genes for vitamin B_1_ synthesis (**S1-S5 Tables in S1 File**) and could provide *Microcystis* with this essential nutrient in natural populations.

### Potential for interaction in the *Microcystis* physcosphere

**Fig 3:**
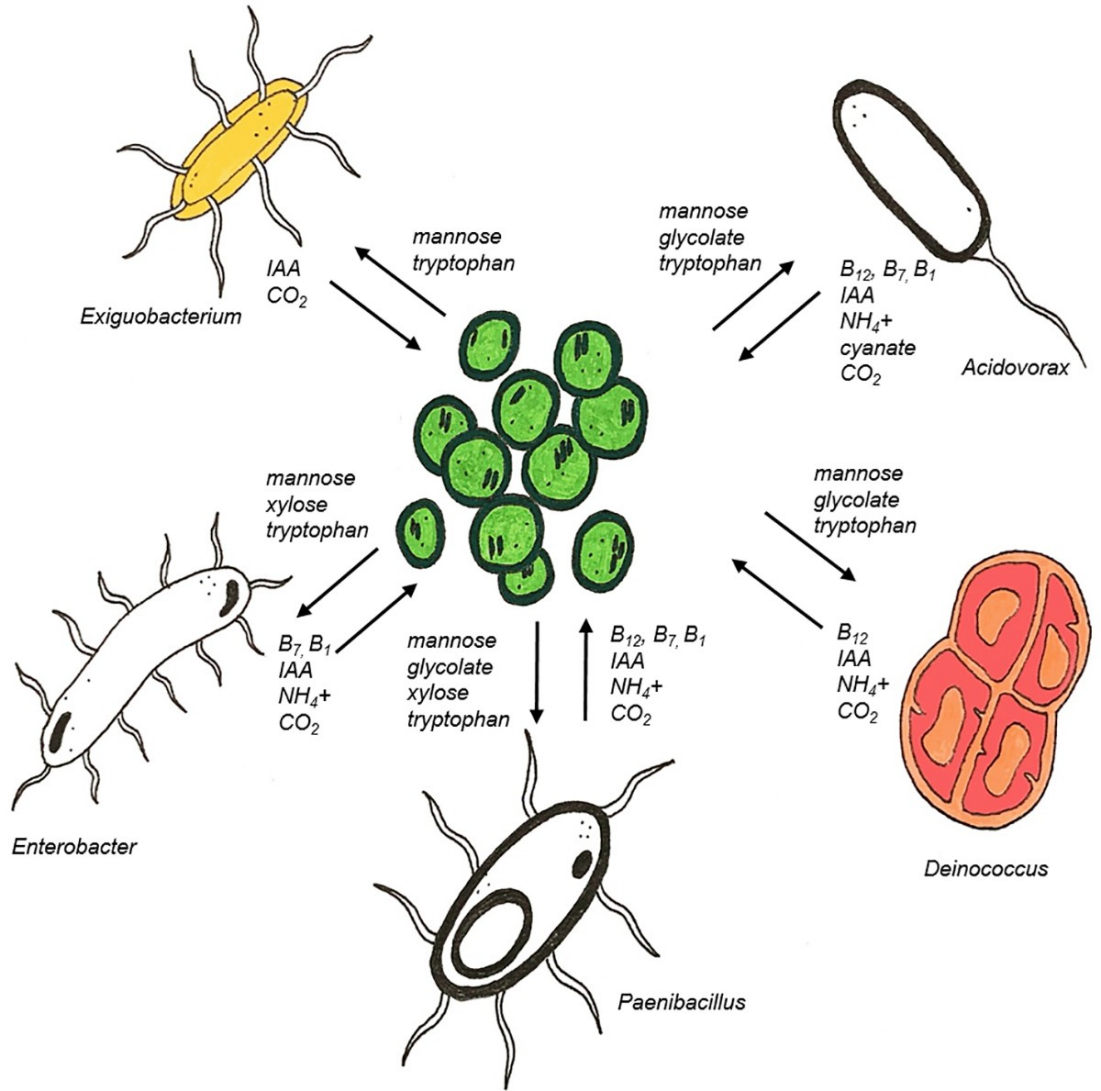
Proposed mechanisms of interaction in the *Microcystis* phycosphere. The genomes of the five sequenced Lake Erie isolates indicate the genetic potential for bidirectional exchange of nutrients and other compounds with *Microcystis* colonies.

The reductionist approach to understanding the dynamics of HABs is shifting toward a more dynamic model. No organism lives in isolation, including the phytoplankton which form HABs. The phycosphere, a potential hotbed for interactions between algae and their heterotrophic bacterial microbiome, can be considered a counterpart of the terrestrial rhizosphere. Within this microenvironment, exchange of nutrients and other compounds drive the mutualistic relationships between phytoplankton like *Microcystis* and their associated bacteria. Genomic analysis of five bacterial isolates from a 2017 *Microcystis* bloom in Lake Erie indicate these bacteria have the genetic potential for bidirectional exchange of nutrients and other growth-promoting compounds such as vitamins and hormones with their photosynthetic partner. The carbon-rich EPS produced by *Microcystis* contains the sugars mannose and xylose, which can be taken up and utilized by all five of the Lake Erie isolates (**Fig 3**). For decades, it has been posited that the bacteria associated with the *Microcystis* mucilage likely benefit from access to these various forms of carbon [38, 100]. Less is known, however, about how *Microcystis* may benefit from this close association with heterotrophic bacteria. One potentially important mechanism of exchange may be the bidirectional exchange of carbon. Bacteria respire CO_2_, as well as produce it as a byproduct of the hydrolysis of urea. During peak bloom conditions, *Microcystis* populations in Lake Erie have increased transcription of genes involved in carbon concentration, suggesting the potential for CO_2_ limitation in dense bloom populations [101]. Respiration by associated bacteria could provide a supplemental source of CO_2_ for *Microcystis* (**Fig 3**) [102]. Many phytoplankton, including *Microcystis* require an exogenous source of vitamins B_12_, B_1_, and B_7_ [97, 99]. Phycosphere bacteria have been identified as potential sources of these vitamins for marine bacteria, and four of the five Lake Erie isolates may serve in this capacity during *Microcystis* blooms (**Fig 3**). In addition to providing vitamins, these bacteria may also provide reduced N to *Microcystis* during periods of N stress, which is common in systems such as Lake Erie during peak bloom conditions (**Fig 3**). Interestingly, all five of the sequenced Lake Erie isolates can produce the plant hormone IAA when supplemented with tryptophan. IAA has been shown to have growth promoting effects on both freshwater and marine algae [16, 91], and may have an important role in the mutualistic exchange that occurs between *Microcystis* and its associated bacteria (**Fig 3**). The genetic capacity of these bacteria to provide critical vitamins and other nutrients provides new insight into the role that biotic interactions may have in the development of *Microcystis* blooms. Further culture studies will reveal the mechanisms which underlie these hypothesized multidimensional interactions illustrated in the genomic potential of these five bacterial isolates.

## Methods

### Sample collection, isolation, and identification

Water samples were collected from Lake Erie on August 9, 2017 from the Ohio State University Stone Lab R/V Gilbraltar III. Samples were taken from four different stations: WE02 (N 41° 45.777’, W 83° 12.931’), WE04 (N 41° 49.634’, W 83° 11.659’), WE13 (N 41° 44.619’, W 83° 08.081’), and MB18 (N 41° 44.886’, W 83° 24.061’). 20 μm and 80 μm mesh plankton nets were used to collect samples from each site to ensure only those bacteria tightly associated with the *Microcystis* colonies would be isolated. 150 mL were collected and stored on ice until transport back to the laboratory.

Both general (LB agar) and selective (CT medium with 100 μM urea agar) media were used for the isolation of bacterial samples [63]. Bacterial isolates are maintained on CT-TY, CT medium with 1 g/L tryptone and 1 g/L yeast extract [103]. Roughly 500 μL of each water sample was plated onto each medium and incubated at 26°C and 32°C, for 48 hours (LB/CT-TY) or seven days (CT-urea). Single colonies were re-streaked onto new plates of the respective media until the isolates were pure as confirmed by microscopy.

Isolates that were grown on urea-supplemented CT were tested for urea utilization capabilities. Briefly, the isolates were inoculated into urea broth and urea agar slants (Hardy Diagnostics) and observed for color change to indicate urease activity *via* ammonia production. To extract DNA for *ureC* screening, turbid overnight cultures were pelleted at 17949 x *g*. The pellet was resuspended in 500 μL of sterile water and heated at 95°C in a dry bath for 15 minutes. After heating, the tubes were centrifuged again for one minute. The supernatant containing the DNA template was used for PCR amplification of the *ureC* gene using primers IGKAGNP-forward (5’ATHGGIAARGCIGGIAAYCC3’) and HEDWGA-reverse (5’IGYICCCCARTCYTCRTG 3’) (modified from Collier et al. [104]). The PCR program was as follows: 94°C for one minute, 25 cycles of 94°C for 30 seconds, 53°C for 30 seconds, and 72°C for 45 seconds, with a final extension of 72°C for 10 minutes.

### DNA extraction and sequencing

Isolates were grown from a single colony in CT-TY broth for 48 hours at 26°C or 32°C (JMULE4). The DNeasy UltraClean Microbial Kit (Qiagen) was used for DNA extraction according to manufacturer’s instructions. A NanoVue Plus (GE Healthcare) was used to check the quantity and purity of the DNA. The genomic DNA was sent to Genewiz (South Plainfield, NJ, USA) for sequencing on the PacBio Sequel System. The PacBio SMRTbell library was prepared according to the manufacturer’s instructions. The SMRTbell libraries were then sequenced with the PacBio Sequel System.

The SMRTLink suite was used to demultiplex the sequence libraries that were generated from the PacBio Sequel platform. These demultiplexed sequence files (BAM and FASTQ) were then provided by the GENEWIZ sequencing facility for assembly and annotation.

### Genome assembly and annotation

The genomes were assembled using the PacBio *de novo* assembly pipeline on the CLC Genomics Workbench plugin CLC Genome Finishing Module (Qiagen) using default parameters. First, the raw reads were imported into CLC Genomics Workbench and corrected for sequencing errors and untrimmed adapters. The error-corrected reads were then assembled into contigs with the “*de novo* Assemble PacBio Reads” tool. The corrected reads were then mapped to the contigs to close gaps and join contigs and subsequently mapped to the larger contigs.

The genomes were annotated with the NCBI prokaryotic genome annotation pipeline (PGAP) [105] and RAST [106, 107]. The annotated genomes were viewed on the RAST SEED Viewer [50]. Genome assemblies and annotations are available through NCBI at BioProject PRJNA521711 and RAST (6666666.419766—*Exiguobacterium* sp. JMULE1, 6666666.419768—*Enterobacter* sp. JMULE2, 6666666.419773—*Deinococcus* sp. JMULE3, 6666666.419775—*Paenibacillus* sp. JMULE4, 6666666.419779—*Acidovorax* sp. JMULE5). Average nucleotide identity (ANI) was calculated using the calculator at http://enve-omics.ce.gatech.edu/ani/ [42]. Amino acid alignments were generated using T-coffee (http://tcoffee.crg.cat/apps/tcoffee/) and phylogeny was calculated using the Maximum-Likelihood method with a Bootstrap test of phylogeny (1000 iterations) in Mega X [108–110].

### Confirmation of IAA production

Bacterial isolates were tested for the capability to produce the auxin indole-3-acetic acid (IAA). The isolates were inoculated in 1mL of CT-TY broth fortified with 5 mM L-tryptophan and incubated while shaking at 26°C, with the exception of 32°C for *Paenibacillus*, for 48 hours. 200 μL of axenic *Microcystis* and 2 μL of broth containing each isolate were added to a 96-well plate in triplicate. 200 μL of the isolate alone were also added in triplicate to the 96-well plate. The well plate was then left to incubate for 24 hours at 26°C. 150 μL of Salkowski’s reagent (0.5 M FeCl_3_ and 70% perchloric acid) were added to each well in the dark [102]. The plate was incubated in the dark for 30 minutes before measuring absorbance at 530 nm to observe color change and compare to a standard curve.

### Prevalence in environmental data

To determine whether these isolates are present during bloom conditions, we analyzed sequence libraries from blooms that occurred in three locations: Taihu (China) [103], Lake Erie (North America) [4, 103], and Lake Greenfield (North America). Recruitments were performed in CLC Genomics Workbench with a similarity fraction of 0.8 and a length fraction of 0.5 to capture closely related organisms [104]. All recruited reads were then classified via blastn in CLC Genomics Workbench, and those that did not match each isolate at the family- or genus-level based on e-value were excluded. The Lake Greenfield samples were collected on 20 July 2018 from the dock and a second site at a drainage pipe. The samples were collected during a period when the town of Greenfield detected microcystin in the drinking water supply. One liter of water was filtered through Sterivex units and kept on ice until frozen at -20°C (~2 hours). Samples were extracted using the DNEasy® PowerWater® (Qiagen) extraction kit [105]. Genomic DNA was sent to GeneWIZ® for library preparation and sequencing on the Illumina HiSeq4000 platform to generate 150 bp paired end reads. Reads are available at the NCBI SRA under BioProject PRJNA610583.

## Supporting information

S1 File (DOCX)

## References

[1] Brittain SM, Wang J, Babcock-Jackson L, Carmichael WW, Rinehart KL, Culver DA. Isolation and characterization of microcystins, cyclic heptapeptide hepatotoxins from a Lake Erie Strain of *Microcystis aeruginosa*. J Gt Lakes Res. 2000;26: 241–249. doi: 10.1016/S0380-1330(00)70690-3

[2] Harke MJ, Steffen MM, Gobler CJ, Otten TG, Wilhelm SW, Wood SA, et al. A review of the global ecology, genomics, and biogeography of the toxic cyanobacterium, *Microcystis* spp. Harmful Algae Glob Expans Harmful Cyanobacterial Blooms Divers Ecol Causes Controls. 2016;54: 4–20. doi: 10.1016/j.hal.2015.12.007 28073480

[3] Qin B, Zhu G, Gao G, Zhang Y, Li W, Paerl HW, et al. A drinking water crisis in Lake Taihu, China: linkage to climatic variability and lake management. Environ Manage. 2010;45: 105–112. doi: 10.1007/s00267-009-9393-6 19915899

[4] Steffen MM, Davis TW, McKay RM, Bullerjahn GS, Krausfeldt LE, Stough JMA, et al. Ecophysiological examination of the Lake Erie *Microcystis* bloom in 2014: Linkages between biology and the water supply shutdown of Toledo, OH. Environ Sci Technol. 2017;51: 6745–6755. doi: 10.1021/acs.est.7b00856 28535339

[5] Visser PM, Ibelings BW, Bormans M, Huisman J. Artificial mixing to control cyanobacterial blooms: a review. Aquat Ecol. 2016;50: 423–441.

[6] Van Hullebusch E, Deluchat V, Chazal PM, Baudu M. Environmental impact of two successive chemical treatments in a small shallow eutrophied lake: Part II. Case of copper sulfate. Environ Pollut. 2002;120: 627–634. doi: 10.1016/s0269-7491(02)00191-4 12442786

[7] Rastogi RP, Madamwar D, Incharoensakdi A. Bloom dynamics of cyanobacteria and their toxins: environmental health impacts and mitigation strategies. Front Microbiol. 2015;6: 1254. doi: 10.3389/fmicb.2015.01254 26635737 PMC4646972

[8] Welch IM, Barrett P, Gibson MT, Ridge I. Barley straw as an inhibitor of algal growth I: studies in the Chesterfield Canal. J Appl Phycol. 1990;2: 231–239.

[9] Zeng Y, Wang J, Yang C, Ding M, Hamilton PB, Zhang X, et al. A *Streptomyces globisporus* strain kills *Microcystis aeruginosa* via cell-to-cell contact. Sci Total Environ. 2021;769: 144489. doi: 10.1016/j.scitotenv.2020.144489 33465632

[10] Saitou T, Sugiura N, Itayama T, Inamori Y, Matsumura M. Degradation characteristics of microcystins by isolated bacteria from Lake Kasumigaura. J Water Supply Res Technol-AQUA. 2003;52: 13–18.

[11] Ho L, Hoefel D, Saint CP, Newcombe G. Isolation and identification of a novel microcystin-degrading bacterium from a biological sand filter. Water Res. 2007;41: 4685–4695. doi: 10.1016/j.watres.2007.06.057 17640697

[12] Yang F, Zhou Y, Yin L, Zhu G, Liang G, Pu Y. Microcystin-degrading activity of an indigenous bacterial strain *Stenotrophomonas acidaminiphila* MC-LTH2 isolated from Lake Taihu. PLoS One. 2014;9: e86216. doi: 10.1371/journal.pone.0086216 24416455 PMC3887098

[13] Steffen MM, Li Z, Effler TC, Hauser LJ, Boyer GL, Wilhelm SW. Comparative metagenomics of toxic freshwater cyanobacteria bloom communities on two continents. PLoS ONE. 2012;7: e44002. doi: 10.1371/journal.pone.0044002 22952848 PMC3430607

[14] Mou X, Lu X, Jacob J, Sun S, Heath R. Metagenomic identification of bacterioplankton taxa and pathways involved in microcystin degradation in Lake Erie. PLoS One. 2013;8: e61890. doi: 10.1371/journal.pone.0061890 23637924 PMC3634838

[15] Bell W, Mitchell R. Chemotactic and growth responses of marine bacteria to algal extracellular products. Biol Bull. 1972;143: 265–277. doi: 10.2307/1540052

[16] Amin SA, Hmelo LR, van Tol HM, Durham BP, Carlson LT, Heal KR, et al. Interaction and signalling between a cosmopolitan phytoplankton and associated bacteria. Nature. 2015;522: 98–101. doi: 10.1038/nature14488 26017307

[17] Seymour JR, Amin SA, Raina J-B, Stocker R. Zooming in on the phycosphere: the ecological interface for phytoplankton–bacteria relationships. Nat Microbiol. 2017;2: 17065. doi: 10.1038/nmicrobiol.2017.65 28555622

[18] Shirai M, Matumaru K, Ohotake A, Takamura Y, Aida T, Nakano M. Development of a solid medium for growth and isolation of axenic *Microcystis* strains (Cyanobacteria). Appl Environ Microbiol. 1989;55: 2569–2571. doi: 10.1128/aem.55.10.2569-2571.1989 16348030 PMC203123

[19] Dziallas C, Grossart H-P. Microbial interactions with the cyanobacterium *Microcystis aeruginosa* and their dependence on temperature. Mar Biol. 2012;159: 2389–2398. doi: 10.1007/s00227-012-1927-4

[20] Kim M, Shin B, Lee J, Park HY, Park W. Culture-independent and culture-dependent analyses of the bacterial community in the phycosphere of cyanobloom-forming *Microcystis aeruginosa*. Sci Rep. 2019;9: 20416. doi: 10.1038/s41598-019-56882-1 31892695 PMC6938486

[21] Wang W, Shen H, Shi P, Chen J, Ni L, Xie P. Experimental evidence for the role of heterotrophic bacteria in the formation of *Microcystis* colonies. J Appl Phycol. 2016;28: 1111–1123. doi: 10.1007/s10811-015-0659-5

[22] Cai H, Jiang H, Krumholz LR, Yang Z. Bacterial community composition of size-fractioned aggregates within the phycosphere of cyanobacterial blooms in a eutrophic freshwater lake. PLOS ONE. 2014;9: e102879. doi: 10.1371/journal.pone.0102879 25144467 PMC4140718

[23] Cook KV, Li C, Cai H, Krumholz LR, Hambright KD, Paerl HW, et al. The global *Microcystis* interactome. Limnol Oceanogr. 2020;65: S194–S207. doi: 10.1002/lno.11361 32051648 PMC7003799

[24] Boedecker AR, Niewinski DN, Newell SE, Chaffin JD, McCarthy MJ. Evaluating sediments as an ecosystem service in western Lake Erie via quantification of nutrient cycling pathways and selected gene abundances. J Gt Lakes Res. 2020;46: 920–932. doi: 10.1016/j.jglr.2020.04.010

[25] Akins L, Ortiz J, Leff LG. Strain-specific responses of toxic and non-toxic *Microcystis aeruginosa* to exudates of heterotrophic bacteria. Hydrobiologia. 2019. doi: 10.1007/s10750-019-04073-4

[26] Wu Q, Zhang X, Jia S, Li J, Li P. Effects of the cultivable bacteria attached to *Microcystis* colonies on the colony size and growth of *Microcystis*. J Freshw Ecol. 2019;34: 663–673. doi: 10.1080/02705060.2019.1665115

[27] S K., S P., S S., M A.B., S G. Removal of microcystin-RR, a membrane foulant using exocellular polymer from *Enterobacter ludwigii*: Kinetic and isotherm studies. Desalination. 2015;369: 175–187. doi: 10.1016/j.desal.2015.05.008

[28] Xu L, Zhou M, Ju H, Zhang Z, Zhang J, Sun C. *Enterobacter aerogenes* metabolites enhance *Microcystis aeruginosa* biomass recovery for sustainable bioflocculant and biohydrogen production. Sci Total Environ. 2018;634: 488–496. doi: 10.1016/j.scitotenv.2018.03.327 29635192

[29] Shia L, Cai Y, Wang X, Li P, Yu Y, Kong F. Community structure of bacteria associated with *Microcystis* colonies from cyanobacterial blooms. null. 2010;25: 193–203. doi: 10.1080/02705060.2010.9665068

[30] Berg KA, Lyra C, Sivonen K, Paulin L, Suomalainen S, Tuomi P, et al. High diversity of cultivable heterotrophic bacteria in association with cyanobacterial water blooms. ISME J. 2008;3: 314–325. doi: 10.1038/ismej.2008.110 19020559

[31] Oh H-M, Lee SJ, Park M-H, Kim H-S, Kim H-C, Yoon J-H, et al. Harvesting of *Chlorella vulgaris* using a bioflocculant from *Paenibacillus* sp. AM49. Biotechnol Lett. 2001;23: 1229–1234. doi: 10.1023/A:1010577319771

[32] Powell RJ, Hill RT. Rapid aggregation of biofuel-producing algae by the bacterium *Bacillus* sp. strain RP1137. Appl Environ Microbiol. 2013;79: 6093–6101. doi: 10.1128/AEM.01496-13 23892750 PMC3811378

[33] Chun S-J, Cui Y, Ko S-R, Lee H-G, Srivastava A, Oh H-M, et al. *Acidovorax lacteus* sp. nov., isolated from a culture of a bloom-forming cyanobacterium (*Microcystis* sp.). Antonie Van Leeuwenhoek. 2017;110: 1199–1205. doi: 10.1007/s10482-017-0892-9 28553696

[34] Krishnan A, Zhang Y-Q, Mou X. Isolation and characterization of microcystin-degrading bacteria from Lake Erie. Bull Environ Contam Toxicol. 2018;101: 617–623. doi: 10.1007/s00128-018-2468-4 30368574

[35] Jankowiak JG, Gobler CJ. The composition and function of microbiomes within *Microcystis* colonies are significantly different than native bacterial assemblages in two North American lakes. Front Microbiol. 2020;11: 1016. doi: 10.3389/fmicb.2020.01016 32547511 PMC7270213

[36] Li Q, Lin F, Yang C, Wang J, Lin Y, Shen M, et al. A large-scale comparative metagenomic study reveals the functional interactions in six bloom-forming *Microcystis*-epibiont communities. Front Microbiol. 2018;9: 746. doi: 10.3389/fmicb.2018.00746 29731741 PMC5919953

[37] Sharma AK, Zhaxybayeva O, Papke RT, Doolittle WF. Actinorhodopsins: proteorhodopsin-like gene sequences found predominantly in non-marine environments. Environ Microbiol. 2008;10: 1039–1056. doi: 10.1111/j.1462-2920.2007.01525.x 18218036

[38] Brunberg A-K. Contribution of bacteria in the mucilage of *Microcystis* spp. (Cyanobacteria) to benthic and pelagic bacterial production in a hypereutrophic lake. FEMS Microbiol Ecol. 1999;29: 13–22. doi: 10.1111/j.1574-6941.1999.tb00594.x

[39] Tarao M, Jezbera J, Hahn MW. Involvement of cell surface structures in size-independent grazing resistance of freshwater Actinobacteria. Appl Environ Microbiol. 2009;75: 4720. doi: 10.1128/AEM.00251-09 19502450 PMC2708440

[40] Ohtsubo Y, Maruyama F, Mitsui H, Nagata Y, Tsuda M. Complete genome sequence of *Acidovorax* sp. strain KKS102, a polychlorinated-biphenyl degrader. J Bacteriol. 2012;194: 6970–6971. doi: 10.1128/JB.01848-12 23209225 PMC3510582

[41] Lau YY, Yin W-F, Chan K-G. *Enterobacter asburiae* strain L1: Complete genome and whole genome optical mapping analysis of a quorum sensing bacterium. Sensors. 2014;14. doi: 10.3390/s140813913 25196111 PMC4178997

[42] Goris J, Konstantinidis KT, Klappenbach JA, Coenye T, Vandamme P, Tiedje JM. DNA–DNA hybridization values and their relationship to whole-genome sequence similarities. Int J Syst Evol Microbiol. 2007;57: 81–91. doi: 10.1099/ijs.0.64483-0 17220447

[43] Rodriguez-R LM, Konstantinidis KT. Bypassing cultivation to identify bacterial species. Microbe. 2014;9: 111–118.

[44] Tang J, Zhang Y, Meng H, Xue Z, Ma J. Complete genome sequence of *Exiguobacterium* sp. strain MH3, isolated from rhizosphere of *Lemna minor*. Genome Announc. 2013;1: 1059. doi: 10.1128/genomeA.01059-13 24356831 PMC3868855

[45] Tang J, Zhang Y, Cui Y, Ma J. Effects of a rhizobacterium on the growth of and chromium remediation by *Lemna minor*. Environ Sci Pollut Res. 2015;22: 9686–9693. doi: 10.1007/s11356-015-4138-y 25631740

[46] Wang J, Davaadelger B, Salazar JK, Butler RR, Pombert J-F, Kilbane JJ, et al. Isolation and characterization of an interactive culture of two *Paenibacillus* species with moderately thermophilic desulfurization ability. Biotechnol Lett. 2015;37: 2201–2211. doi: 10.1007/s10529-015-1918-x 26209032

[47] Butler RR, Wang J, Stark BC, Pombert J-F. Complete genome sequences of two interactive moderate thermophiles, *Paenibacillus napthalenovorans* 32O-Y and *Paenibacillus* sp. 32O-W. Genome Announc. 2016;4: 1717. doi: 10.1128/genomeA.01717-15 26868401 PMC4751325

[48] Xie M, Ren M, Yang C, Yi H, Li Z, Li T, et al. Metagenomic analysis reveals symbiotic relationship among bacteria in *Microcystis*-dominated community. Front Microbiol. 2016;7: 56. doi: 10.3389/fmicb.2016.00056 26870018 PMC4735357

[49] Overbeek R, Begley T, Butler RM, Choudhuri JV, Chuang H-Y, Cohoon M, et al. The subsystems approach to genome annotation and its use in the project to annotate 1000 genomes. Nucleic Acids Res. 2005;33: 5691–5702. doi: 10.1093/nar/gki866 16214803 PMC1251668

[50] Overbeek R, Olson R, Pusch GD, Olsen GJ, Davis JJ, Disz T, et al. The SEED and the Rapid Annotation of microbial genomes using Subsystems Technology (RAST). Nucleic Acids Res. 2013;42: D206–D214. doi: 10.1093/nar/gkt1226 24293654 PMC3965101

[51] Sakka M, Higashi Y, Kimura T, Ratanakhanokchai K, Sakka K. Characterization of *Paenibacillus curdlanolyticus* B-6 Xyn10D, a xylanase that contains a family 3 carbohydrate-binding module. Appl Environ Microbiol. 2011;77: 4260. doi: 10.1128/AEM.00226-11 21498754 PMC3131646

[52] Sermsathanaswadi J, Baramee S, Tachaapaikoon C, Pason P, Ratanakhanokchai K, Kosugi A. The family 22 carbohydrate-binding module of bifunctional xylanase/β-glucanase Xyn10E from *Paenibacillus curdlanolyticus* B-6 has an important role in lignocellulose degradation. Enzyme Microb Technol. 2017;96: 75–84. doi: 10.1016/j.enzmictec.2016.09.015 27871388

[53] Arandia-Gorostidi N, Weber P K, Alonso-Saez L, Moran XA G, Mayali X. Elevated temperature increases carbon and nitrogen fluxes between phytoplankton and heterotrophic bacteria through physical attachment. ISME J. 2016. Available: doi: 10.1038/ismej.2016.156 27922602 PMC5322308

[54] Van den M, Middelburg JJ, Soetaert K, van Rijswijk P, Boschker HTS, Heip CHR. Carbon-nitrogen coupling and algal-bacterial interactions during an experimental bloom: Modeling a 13C tracer experiment. Limnol Oceanogr. 2004;49: 862–878. doi: 10.4319/lo.2004.49.3.0862

[55] Fallon R.D., Brock T.D. Decomposition of blue-green algal (cyanobacterial) blooms in Lake Mendota, Wisconsin. Appl Environ Microbiol. 1979:37: 820. doi: 10.1128/aem.37.5.820-830.1979 16345380 PMC243308

[56] Kamennaya NA, Chernihovsky M, Post AF. The cyanate utilization capacity of marine unicellular Cyanobacteria. Limnol Oceanogr. 2008;53: 2485–2494. doi: 10.4319/lo.2008.53.6.2485

[57] Harke MJ, Davis TW, Watson SB, Gobler CJ. Nutrient-controlled niche differentiation of western Lake Erie cyanobacterial populations revealed via metatranscriptomic surveys. Environ Sci Technol. 2016;50: 604–615. doi: 10.1021/acs.est.5b03931 26654276

[58] Yang C, Wang Q, Simon PN, Liu J, Liu L, Dai X, et al. Distinct network interactions in particle-associated and free-living bacterial communities during a *Microcystis aeruginosa* bloom in a plateau lake. Front Microbiol. 2017;8. doi: 10.3389/fmicb.2017.01202 28713340 PMC5492469

[59] Fernandes G de C, Trarbach LJ, de Campos SB, Beneduzi A, Passaglia LMP. Alternative nitrogenase and pseudogenes: unique features of the *Paenibacillus riograndensis* nitrogen fixation system. Res Microbiol. 2014;165: 571–580. doi: 10.1016/j.resmic.2014.06.002 24956360

[60] Liu X, Li Q, Li Y, Guan G, Chen S. *Paenibacillus* strains with nitrogen fixation and multiple beneficial properties for promoting plant growth. PeerJ. 2019;7: e7445. doi: 10.7717/peerj.7445 31579563 PMC6761918

[61] Lyons EM, Thiel T. Characterization of *nifB*, *nifS*, and *nifU* genes in the cyanobacterium *Anabaena variabilis*: *nifB* is required for the vanadium-dependent nitrogenase. J Bacteriol. 1995;177: 1570–1575. doi: 10.1128/jb.177.6.1570-1575.1995 7883714 PMC176774

[62] Mobley HL, Hausinger RP. Microbial ureases: significance, regulation, and molecular characterization. Microbiol Rev. 1989;53: 85–108. doi: 10.1128/mr.53.1.85-108.1989 2651866 PMC372718

[63] Steffen MM, Dearth SP, Dill BD, Li Z, Larsen KM, Campagna SR, et al. Nutrients drive transcriptional changes that maintain metabolic homeostasis but alter genome architecture in *Microcystis*. ISME J. 2014;8: 2080–2092. doi: 10.1038/ismej.2014.78 24858783 PMC4184021

[64] Krausfeldt LE, Farmer AT, Castro Gonzalez HF, Zepernick BN, Campagna SR, Wilhelm SW. Urea is both a carbon and nitrogen source for *Microcystis aeruginosa*: Tracking 13C incorporation at bloom pH conditions. Front Microbiol. 2019;10: 1064. doi: 10.3389/fmicb.2019.01064 31164875 PMC6536089

[65] Yamamoto Y, Nakahara H. Competitive dominance of the cyanobacterium *Microcystis aeruginosa* in nutrient-rich culture conditions with special reference to dissolved inorganic carbon uptake. Phycol Res. 2005;53: 201–208. doi: 10.1111/j.1440-183.2005.00387.x

[66] Dixon NE, Gazzola C, Blakeley RL, Zerner B. Jack bean urease (EC 3.5.1.5). Metalloenzyme. Simple biological role for nickel. J Am Chem Soc. 1975;97: 4131–4133. doi: 10.1021/ja00847a045 1159216

[67] Boer JL, Mulrooney SB, Hausinger RP. Nickel-dependent metalloenzymes. Arch Biochem Biophys. 2014;544: 142–152. doi: 10.1016/j.abb.2013.09.002 24036122 PMC3946514

[68] Cho BC, Park MG, Shim JH, Azam F. Significance of bacteria in urea dynamics in coastal surface waters. Mar Ecol Prog Ser. 1996;142: 19–26.

[69] Michihiko Nakagawa, Takamura Y, Yagi O. Isolation and characterization of the slime from a cyanobacterium, *Microcystis aeruginosa* K-3A. Agric Biol Chem. 1987;51: 329–337. doi: 10.1271/bbb1961.51.329

[70] Forni C, Telo’ FR, Caiola MG. Comparative analysis of the polysaccharides produced by different species of *Microcystis* (Chroococcales, Cyanophyta). Phycologia. 1997;36: 181–185. doi: 10.2216/i0031-8884-36-3-181.1

[71] Weiss G, Kovalerchick D, Lieman-Hurwitz J, Murik O, De Philippis R, Carmeli S, et al. Increased algicidal activity of *Aeromonas veronii* in response to *Microcystis aeruginosa*: interspecies crosstalk and secondary metabolites synergism. Environ Microbiol. 2019;21: 1140–1150. doi: 10.1111/1462-2920.14561 30761715

[72] Girolod D, Ortolano PI, Vieira AA. Bacteria–algae association in batch cultures of phytoplankton from a tropical reservoir: The significance of algal carbohydrates. Freshw Biol. 2007;52: 1281–1289. doi: 10.1111/j.1365-2427.2007.01764.x

[73] Landa M, Burns AS, Roth SJ, Moran MA. Bacterial transcriptome remodeling during sequential co-culture with a marine dinoflagellate and diatom. ISME J. 2017;11: 2677–2690. doi: 10.1038/ismej.2017.117 28731474 PMC5702724

[74] Zheng Q, Lu J, Wang Y, Jiao N. Genomic reconstructions and potential metabolic strategies of generalist and specialist heterotrophic bacteria associated with an estuary *Synechococcus* culture. FEMS Microbiol Ecol. 2019;95: fiz017. doi: 10.1093/femsec/fiz017 30689834

[75] Hellebust JA. Excretion of some organic compounds by marine phytoplankton. Limnol Oceanogr. 1965;10: 192–206.

[76] Lau WWY, Keil RG, Armbrust EV. Succession and diel transcriptional response of the glycolate-utilizing component of the bacterial community during a spring phytoplankton bloom. Appl Environ Microbiol. 2007;73: 2440. doi: 10.1128/AEM.01965-06 17293517 PMC1855595

[77] Paver SF, Kent AD. Temporal patterns in glycolate-utilizing bacterial community composition correlate with phytoplankton population dynamics in humic lakes. Microb Ecol. 2010;60: 406–418. doi: 10.1007/s00248-010-9722-6 20652236

[78] Lau WWY, Armbrust EV. Detection of glycolate oxidase gene *glcD* diversity among cultured and environmental marine bacteria. Environ Microbiol. 2006;8: 1688–1702. doi: 10.1111/j.1462-2920.2006.01092.x 16958750

[79] Woodhouse JN, Kinsela AS, Collins RN, Bowling LC, Honeyman GL, Holliday JK, et al. Microbial communities reflect temporal changes in cyanobacterial composition in a shallow ephemeral freshwater lake. Isme J. 2015;10: 1337. doi: 10.1038/ismej.2015.218 26636552 PMC5029192

[80] Reynoso G, Smith MR, Holmes CP, Keelan CR, McGrath SE, Alvarez GH, et al. Bacterial community structure and response to nitrogen amendments in Lake Shenandoah (VA, USA). Water Sci Technol. 2019;80: 675–684. doi: 10.2166/wst.2019.311 31661447

[81] Xing W, Huang W, Li D, Liu Y. Effects of iron on growth, pigment content, photosystem II efficiency, and siderophores production of *Microcystis aeruginosa* and *Microcystis wesenbergii*. Curr Microbiol. 2007;55: 94–98. doi: 10.1007/s00284-006-0470-2 17632756

[82] Fischbach MA, Lin H, Liu DR, Walsh CT. How pathogenic bacteria evade mammalian sabotage in the battle for iron. Nat Chem Biol. 2006;2: 132–138. doi: 10.1038/nchembio771 16485005

[83] Williams PH. Novel iron uptake system specified by ColV plasmids: an important component in the virulence of invasive strains of *Escherichia coli*. Infect Immun. 1979;26: 925–932. doi: 10.1128/iai.26.3.925-932.1979 160892 PMC414708

[84] Goldman SJ, Lammers PJ, Berman MS, Sanders-Loehr J. Siderophore-mediated iron uptake in different strains of *Anabaena* sp. J Bacteriol. 1983;156: 1144–1150. doi: 10.1128/jb.156.3.1144-1150.1983 6227608 PMC217960

[85] Yokoo S, Inoue S, Suzuki N, Amakawa N, Matsui H, Nakagami H, et al. Comparative analysis of plant isochorismate synthases reveals structural mechanisms underlying their distinct biochemical properties. Biosci Rep. 2018;38: BSR20171457. doi: 10.1042/BSR20171457 29436485 PMC5843753

[86] Wildermuth MC, Dewdney J, Wu G, Ausubel FM. Isochorismate synthase is required to synthesize salicylic acid for plant defence. Nature. 2001;414: 562–565. doi: 10.1038/35107108 11734859

[87] Ollinger J, Song K-B, Antelmann H, Hecker M, Helmann JD. Role of the fur regulon in iron transport in *Bacillus subtilis*. J Bacteriol. 2006;188: 3664–3673. doi: 10.1128/JB.188.10.3664-3673.2006 16672620 PMC1482855

[88] Wilson MK, Abergel RJ, Raymond KN, Arceneaux JEL, Byers BR. Siderophores of *Bacillus anthracis*, *Bacillus cereus*, and *Bacillus thuringiensis*. Biochem Biophys Res Commun. 2006;348: 320–325. doi: 10.1016/j.bbrc.2006.07.055 16875672

[89] Hertlein G, Müller S, Garcia-Gonzalez E, Poppinga L, Süssmuth RD, Genersch E. Production of the catechol type siderophore bacillibactin by the honey bee pathogen *Paenibacillus larvae*. PLOS ONE. 2014;9: e108272. doi: 10.1371/journal.pone.0108272 25237888 PMC4169593

[90] Braun V. Energy-coupled transport and signal transduction through the Gram-negative outer membrane via TonB-ExbB-ExbD-dependent receptor proteins. FEMS Microbiol Rev. 1995;16: 295–307. doi: 10.1111/j.1574-6976.1995.tb00177.x 7654405

[91] Lee C, Jeon MS, Kim JY, Lee SH, Kim DG, Roh SW, et al. Effects of an auxin-producing symbiotic bacterium on cell growth of the microalga *Haematococcus pluvialis*: Elevation of cell density and prolongation of exponential stage. Algal Res. 2019;41: 101547. doi: 10.1016/j.algal.2019.101547

[92] Kaneko T, Nakajima N, Okamoto S, Suzuki I, Tanabe Y, Tamaoki M, et al. Complete genomic structure of the bloom-forming toxic cyanobacterium *Microcystis aeruginosa* NIES-843. DNA Res. 2007;14: 247–256. doi: 10.1093/dnares/dsm026 18192279 PMC2779907

[93] Federle MJ. Autoinducer-2-based chemical communication in bacteria: Complexities of interspecies signaling. Contrib Microbiol. 2009;16: 18–32. doi: 10.1159/000219371 19494577 PMC3042238

[94] Vendeville A, Winzer K, Heurlier K, Tang CM, Hardie KR. Making “sense” of metabolism: autoinducer-2, LUXS and pathogenic bacteria. Nat Rev Microbiol. 2005;3: 383–396. doi: 10.1038/nrmicro1146 15864263

[95] Xavier KB, Bassler BL. Regulation of uptake and processing of the quorum-sensing autoinducer AI-2 in *Escherichia coli*. J Bacteriol. 2005;187: 238–248. doi: 10.1128/JB.187.1.238-248.2005 15601708 PMC538819

[96] Wilhelm SW, Trick CG. Effects of vitamin B12 concentration on chemostat cultured *Synechococcus* sp. strain PCC 7002. Can J Microbiol. 1995;41: 145–151. doi: 10.1139/m95-019

[97] Croft MT, Lawrence AD, Raux-Deery E, Warren MJ, Smith AG. Algae acquire vitamin B12 through a symbiotic relationship with bacteria. Nature. 2005;438: 90–93. doi: 10.1038/nature04056 16267554

[98] Kazamia E, Czesnick H, Nguyen TTV, Croft MT, Sherwood E, Sasso S, et al. Mutualistic interactions between vitamin B12-dependent algae and heterotrophic bacteria exhibit regulation. Environ Microbiol. 2012;14: 1466–1476. doi: 10.1111/j.1462-2920.2012.02733.x 22463064

[99] Tang YZ, Koch F, Gobler CJ. Most harmful algal bloom species are vitamin B1 and B12 auxotrophs. Proc Natl Acad Sci. 2010;107: 20756–20761. doi: 10.1073/pnas.1009566107 21068377 PMC2996436

[100] Worm J, Søndergaard M. Dynamics of heterotrophic bacteria attached to *Microcystis* spp. (Cyanobacteria). Aquat Microb Ecol. 1998;14: 19–28. doi: 10.3354/ame014019

[101] Steffen MM, Belisle BS, Watson SB, Boyer GL, Bourbonniere RA, Wilhelm SW. Metatranscriptomic evidence for co-occurring top-down and bottom-up controls on toxic cyanobacterial communities. Appl Environ Microbiol. 2015;81: 3268–3276. doi: 10.1128/AEM.04101-14 25662977 PMC4393433

[102] Kouzuma A, Watanabe K. Exploring the potential of algae/bacteria interactions. Curr Opin Biotechnol. 2015;33: 125–129. doi: 10.1016/j.copbio.2015.02.007 25744715

[103] Harvey EL, Deering RW, Rowley DC, El Gamal A, Schorn M, Moore BS, et al. A bacterial quorum-sensing precursor induces mortality in the marine coccolithophore, *Emiliania huxleyi*. Front Microbiol. 2016;7: 59. doi: 10.3389/fmicb.2016.00059 26870019 PMC4737879

[104] Collier JL, Baker KM, Bell SL. Diversity of urea-degrading microorganisms in open-ocean and estuarine planktonic communities. Environ Microbiol. 2009;11: 3118–3131. doi: 10.1111/j.1462-2920.2009.02016.x 19659552

[105] Tatusova T, DiCuccio M, Badretdin A, Chetvernin V, Nawrocki EP, Zaslavsky L, et al. NCBI prokaryotic genome annotation pipeline. Nucleic Acids Res. 2016;44: 6614–6624. doi: 10.1093/nar/gkw569 27342282 PMC5001611

[106] Aziz RK, Bartels D, Best AA, DeJongh M, Disz T, Edwards RA, et al. The RAST Server: Rapid annotations using subsystems technology. BMC Genomics. 2008;9: 75. doi: 10.1186/1471-2164-9-75 18261238 PMC2265698

[107] Brettin T, Davis JJ, Disz T, Edwards RA, Gerdes S, Olsen GJ, et al. RASTtk: A modular and extensible implementation of the RAST algorithm for building custom annotation pipelines and annotating batches of genomes. Sci Rep. 2015;5: 8365. doi: 10.1038/srep08365 25666585 PMC4322359

[108] Notredame C, Higgins DG, Heringa J. T-coffee: a novel method for fast and accurate multiple sequence alignment11Edited by J. Thornton. J Mol Biol. 2000;302: 205–217. doi: 10.1006/jmbi.2000.4042 10964570

[109] Di Tommaso P, Moretti S, Xenarios I, Orobitg M, Montanyola A, Chang J-M, et al. T-Coffee: a web server for the multiple sequence alignment of protein and RNA sequences using structural information and homology extension. Nucleic Acids Res. 2011;39: W13–W17. doi: 10.1093/nar/gkr245 21558174 PMC3125728

[110] Kumar S, Stecher G, Li M, Knyaz C, Tamura K. MEGA X: Molecular evolutionary genetics analysis across computing platforms. Mol Biol Evol. 2018;35: 1547–1549. doi: 10.1093/molbev/msy096 29722887 PMC5967553

