# Supplementary material for: Genomic signatures of Lake Erie bacteria suggest interaction in the Microcystis phycosphere
Source: PLoS One. 2021 Sep 22;16(9):e0257017. doi: 10.1371/journal.pone.0257017 (PMC8457463; doi:10.1371/journal.pone.0257017)
Supplement: S1 File — (DOCX) [file pone.0257017.s001.docx]

**S1 File**

**S1 Table.** The genomic DNA concentration, quality (260/280), and resultant total number of raw reads for each isolate, with the number of reads in parentheses after the sequences were corrected in the PacBio *de novo* assembly pipeline.

| Isolate | DNA (ng/µL) | 260/280 | Total BP | Total Reads |
| --- | --- | --- | --- | --- |
| *Exiguobacterium* sp. JMULE1 | 462.8 | 1.91 | 1,297,742,008 | 334,339 (49,904) |
| *Enterobacter* sp. JMULE2 | 479.9 | 1.90 | 1,818,930,107 | 455,299 (69,289) |
| *Deinococcus* sp. JMULE3 | 693.0 | 1.93 | 831,129,345 | 197,286 (27,959) |
| *Paenibacillus* sp. JMULE4 | 176.4 | 1.89 | 1,422,433,251 | 378,793 (56,789) |
| *Acidovorax* sp. JMULE5 | 459.9 | 1.92 | 1,490,700,321 | 354,329 (50,990) |

**S2 Table.** Locus tags for genes of interest in the genome of the reported Lake Erie spp. as annotated by RAST/PGAP.

| Organism | Gene | Locus tag | Function/Product | Subsystem |
| --- | --- | --- | --- | --- |
| *Acidovorax* |  | EXV95_03450 | LysR family transcriptional regulator (PGAP), Cyn operon transcriptional activator (RAST) | Cyanate hydrolysis |
| *Acidovorax* |  | EXV95_19915 | Nitrate ABC transporter substrate-binding protein (PGAP), Cyanate ABC transporter (RAST) | Cyanate hydrolysis |
| *Acidovorax* |  | EXV95_19925 | ABC transporter ATP-binding protein | Cyanate hydrolysis |
| *Acidovorax* | *cynS* | EXV95_19930 | Cyanase (PGAP), Cyanate hydratase (RAST) | Cyanate hydrolysis |
| *Acidovorax* | *ntrB* | EXV95_19920 | Nitrate ABC transporter, permease protein | Cyanate hydrolysis |
| *Acidovorax* | *norR* | EXV95_17390 | Nitric oxide reductase transcriptional regulator NorR | Nitrosative stress |
| *Acidovorax* |  | EXV95_12385 | NnrS family protein | Nitrosative stress |
| *Acidovorax* |  | EXV95_01575 | Rrf2 family transcriptional regulator (PGAP), Nitrite-sensitive transcriptional repressor NsrR (RAST) | Nitrosative stress |
| *Acidovorax* |  | EXV95_17395 | Nitric-oxide reductase large subunit | Nitrosative stress |
| *Acidovorax* |  | EXV95_19325 | Nitrate reductase subunit alpha | Nitrate and nitrite ammonification |
| *Acidovorax* |  | EXV95_24715 | ANTAR domain-containing protein (PGAP), Response regulator NasT (RAST) | Nitrate and nitrite ammonification |
| *Acidovorax* |  | EXV95_24640 | NarK/NasA family nitrate transporter | Nitrate and nitrite ammonification |
| *Acidovorax* |  | EXV95_24700 | ABC transporter ATP-binding protein | Nitrate and nitrite ammonification |
| *Acidovorax* | *narI* | EXV95_19340 | Respiratory nitrate reductase subunit gamma | Nitrate and nitrite ammonification |
| *Acidovorax* | *ntrB* | EXV95_24705 | Nitrate ABC transporter, permease protein | Nitrate and nitrite ammonification |
| *Acidovorax* | *narH* | EXV95_19330 | Nitrate reductase subunit beta | Nitrate and nitrite ammonification |
| *Acidovorax* | *narJ* | EXV95_19335 | Nitrate reductase molybdenum cofactor assembly chaperone (PGAP), Respiratory nitrate reductase delta chain (RAST) | Nitrate and nitrite ammonification |
| *Acidovorax* |  | EXV95_19280 | HAMP domain-containing protein (PGAP), Nitrate/nitrite sensor protein (RAST) | Nitrate and nitrite ammonification |
| *Acidovorax* |  | EXV95_19285 | Response regulator | Nitrate and nitrite ammonification |
| *Acidovorax* | *nirB* | EXV95_07010 | Nitrite reductase large subunit | Nitrate and nitrite ammonification |
| *Acidovorax* | *nirD* | EXV95_07005 | Nitrite reductase small subunit NirD | Nitrate and nitrite ammonification |
| *Acidovorax* |  | EXV95_14455 | FMN-binding glutamate synthase family protein | Ammonia assimilation |
| *Acidovorax* | *glnK* | EXV95_17845 | P-II family nitrogen regulator | Ammonia assimilation |
| *Acidovorax* |  | EXV95_14585 | Class II glutamine amidotransferase | Ammonia assimilation |
| *Acidovorax* | *glnE* | EXV95_15620 | Bifunctional [glutamate--ammonia ligase]-adenylyl-L-tyrosine phosphorylase/[glutamate--ammonia-ligase] adenylyltransferase | Ammonia assimilation |
| *Acidovorax* |  | EXV95_09225 | Ammonium transporter | Ammonia assimilation |
| *Acidovorax* |  | EXV95_21865 | Glutamate synthase subunit alpha | Ammonia assimilation |
| *Acidovorax* |  | EXV95_02385 | [Protein-PII] uridylyltransferase | Ammonia assimilation |
| *Acidovorax* | *glnA* | EXV95_08060 | Type I glutamate--ammonia ligase | Ammonia assimilation |
| *Acidovorax* |  | EXV95_21870 | Glutamate synthase subunit beta | Ammonia assimilation |
| *Acidovorax* |  | EXV95_02605 | Allantoin permease (PGAP), Predicted hydroxymethylpyrimidine transporter CytX (RAST) | Thiamin biosynthesis |
| *Acidovorax* |  | EXV95_07520 | Transporter substrate-binding domain-containing protein (PGAP), Hydroxymethylpyrimidine ABC transporter, substrate-binding component (RAST) | Thiamin biosynthesis |
| *Acidovorax* | *thiO* | EXV95_02590 | Glycine oxidase ThiO | Thiamin biosynthesis |
| *Acidovorax* | *thiL* | EXV95_14295 | Thiamine-phosphate kinase | Thiamin biosynthesis |
| *Acidovorax* |  | EXV95_10645 | ABC transporter ATP-binding protein | Thiamin biosynthesis |
| *Acidovorax* |  | EXV95_10650 | ABC transporter permease | Thiamin biosynthesis |
| *Acidovorax* |  | EXV95_02575 | Thiamine phosphate synthase | Thiamin biosynthesis |
| *Acidovorax* | *thiS* | EXV95_02585 | Sulfur carrier protein ThiS | Thiamin biosynthesis |
| *Acidovorax* |  | EXV95_09475 | Tryptophan synthase subunit alpha | Auxin biosynthesis |
| *Acidovorax* | *trpD* | EXV95_14185 | Anthranilate phosphoribosyltransferase | Auxin biosynthesis |
| *Acidovorax* | *trpB* | EXV95_09470 | Tryptophan synthase subunit beta | Auxin biosynthesis |
| *Acidovorax* |  | EXV95_09465 | Phosphoribosylanthranilate isomerase | Auxin biosynthesis |
| *Acidovorax* |  | EXV95_01940 | Copper oxidase | Copper homeostasis |
| *Acidovorax* |  | EXV95_01255 | Cation-translocating P-type ATPase | Copper homeostasis |
| *Acidovorax* |  | EXV95_16710 | Copper chaperone | Copper homeostasis |
| *Acidovorax* |  | EXV95_06030 | Bcr/CflA family efflux MFS transporter | Copper homeostasis |
| *Acidovorax* | *cueR* | EXV95_16705 | Cu(I)-responsive transcriptional regulator | Copper homeostasis |
| *Acidovorax* |  | EXV95_01930 | DUF411 domain-containing protein (PGAP), CopG protein (RAST) | Copper homeostasis |
| *Acidovorax* |  | EXV95_01935 | Hypothetical protein (PGAP), Copper tolerance protein (RAST) | Copper homeostasis |
| *Acidovorax* |  | EXV95_08490 | Transporter (PGAP), Heavy metal RND efflux outer membrane protein, CzcC family (RAST) | Cobalt-zinc-cadmium resistance |
| *Acidovorax* |  | EXV95_18305 | Response regulator | Cobalt-zinc-cadmium resistance |
| *Acidovorax* |  | EXV95_08475 | Efflux RND transporter permease subunit (PGAP), Cobalt-zinc-cadmium resistance protein CzcA (RAST) | Cobalt-zinc-cadmium resistance |
| *Acidovorax* |  | EXV95_00995 | Efflux RND transporter periplasmic adaptor subunit | Cobalt-zinc-cadmium resistance |
| *Acidovorax* |  | EXV95_20055 | Cation transporter (PGAP), Cobalt-zinc-cadmium resistance protein (RAST) | Cobalt-zinc-cadmium resistance |
| *Acidovorax* |  | EXV95_14245 | HAMP domain-containing histidine kinase | Cobalt-zinc-cadmium resistance |
| *Acidovorax* |  | EXV95_02000 | Cd(II)/Pb(II)-responsive transcriptional regulator | Cobalt-zinc-cadmium resistance |
| *Acidovorax* | *gyrB* | EXV95_16335 | DNA topoisomerase (ATP-hydrolyzing) subunit B | Resistance to fluoroquinolones |
| *Acidovorax* | *gyrA* | EXV95_06505 | DNA gyrase subunit A | Resistance to fluoroquinolones |
| *Acidovorax* |  | EXV95_08770 | Type IV DNA topoisomerase subunit B | Resistance to fluoroquinolones |
| *Acidovorax* | *parC* | EXV95_08760 | DNA topoisomerase IV subunit A | Resistance to fluoroquinolones |
| *Acidovorax* |  | EXV95_01595 | Redoxin domain-containing protein (PGAP), Membrane protein, suppressor for copper-sensitivity ScsD (RAST) | Copper homeostasis: copper tolerance |
| *Acidovorax* | *lnt* | EXV95_20375 | Apolipoprotein N-acyltransferase | Copper homeostasis: copper tolerance |
| *Acidovorax* |  | EXV95_10970 | Divalent-cation tolerance protein CutA | Copper homeostasis: copper tolerance |
| *Acidovorax* |  | EXV95_20370 | CBS domain-containing protein (PGAP), Magnesium and cobalt efflux protein CorC (RAST) | Copper homeostasis: copper tolerance |
| *Acidovorax* |  | EXV95_08795 | MBL fold metallo-hydrolase | Beta-lactamase |
| *Acidovorax* |  | EXV95_17445 | Sigma-54-dependent Fis family transcriptional regulator | Zinc resistance |
| *Acidovorax* |  | EXV95_04485 | Chromate resistance protein | Resistance to chromium compounds |
| *Acidovorax* |  | EXV95_04475 | Superoxide dismutase | Resistance to chromium compounds |
| *Acidovorax* |  | EXV95_03910 | Chromate transporter | Resistance to chromium compounds |
| *Acidovorax* |  | EXV95_19325 | Nitrate reductase subunit alpha | Denitrifying reductase gene clusters |
| *Acidovorax* |  | EXV95_12430 | C-type cytochrome (PGAP), Nitric-oxide reductase subunit C (RAST) | Denitrifying reductase gene clusters |
| *Acidovorax* |  | EXV95_12440 | CbbQ/NirQ/NorQ/GpvN family protein | Denitrifying reductase gene clusters |
| *Acidovorax* |  | EXV95_23495 | Hypothetical protein (PGAP), Nitrous oxide reductase maturation protein, outer-membrane lipoprotein NosL (RAST) | Denitrifying reductase gene clusters |
| *Acidovorax* |  | EXV95_12445 | VWA domain-containing protein (PGAP), Nitric oxide reductase activation protein NorD (RAST) | Denitrifying reductase gene clusters |
| *Acidovorax* |  | EXV95_23505 | ABC transporter permease (PGAP), Nitrous oxide reductase maturation transmembrane protein NosY (RAST) | Denitrifying reductase gene clusters |
| *Acidovorax* |  | EXV95_23520 | Regulatory protein NosR | Denitrifying reductase gene clusters |
| *Acidovorax* |  | EXV95_23525 | Nitrous-oxide reductase | Denitrifying reductase gene clusters |
| *Acidovorax* |  | EXV95_19320 | MFS transporter (PGAP), Nitrate/nitrite transporter NarK (RAST) | Denitrifying reductase gene clusters |
| *Acidovorax* |  | EXV95_12435 | Nitric-oxide reductase large subunit | Denitrifying reductase gene clusters |
| *Acidovorax* |  | EXV95_19330 | Nitrate reductase subunit beta | Denitrifying reductase gene clusters |
| *Acidovorax* | *narJ* | EXV95_19335 | Nitrate reductase molybdenum cofactor assembly chaperone | Denitrifying reductase gene clusters |
| *Acidovorax* | *narI* | EXV95_19340 | Respiratory nitrate reductase subunit gamma | Denitrifying reductase gene clusters |
| *Acidovorax* |  | EXV95_23510 | ABC transporter ATP-binding protein (PGAP), Nitrous oxide reductase maturation protein NosF (ATPase) (RAST) | Denitrifying reductase gene clusters |
| *Acidovorax* | *nosD* | EXV95_23515 | Nitrous oxide reductase family maturation protein NosD | Denitrifying reductase gene clusters |
| *Acidovorax* |  | EXV95_12385 | NnrS family protein | Denitrification |
| *Acidovorax* |  | EXV95_12430 | C-type cytochrome (PGAP), Nitric-oxide reductase subunit C (RAST) | Denitrification |
| *Acidovorax* |  | EXV95_12435 | Nitric-oxide reductase large subunit | Denitrification |
| *Acidovorax* |  | EXV95_17395 | Nitric-oxide reductase large subunit | Denitrification |
| *Acidovorax* |  | EXV95_23525 | Nitrous-oxide reductase | Denitrification |
| *Acidovorax* |  | EXV95_10530 | Crp/Fnr family transcriptional regulator | Denitrification |
| *Acidovorax* |  | EXV95_23520 | Regulatory protein NosR | Denitrification |
| *Acidovorax* |  | EXV95_23505 | ABC transporter permease (PGAP), Nitrous oxide reductase maturation transmembrane protein NosY (RAST) | Denitrification |
| *Acidovorax* |  | EXV95_16535 | Protein NrnU | Denitrification |
| *Acidovorax* |  | EXV95_23510 | ABC transporter ATP-binding protein | Denitrification |
| *Acidovorax* | *nosD* | EXV95_23515 | Nitrous oxide reductase family maturation protein NosD | Denitrification |
| *Acidovorax* |  | EXV95_12445 | VWA domain-containing protein (PGAP), Nitric oxide reductase activation protein NorD (RAST) | Denitrification |
| *Acidovorax* |  | EXV95_23495 | Hypothetical protein (PGAP), Nitrous oxide reductase maturation protein, outer-membrane lipoprotein NosL (RAST) | Denitrification |
| *Acidovorax* |  | EXV95_12440 | CbbQ/NirQ/NorQ/GpvN family protein | Denitrification |
| *Acidovorax* |  | EXV95_22950 | Bifunctional aminodeoxychorismate synthase component I/aminotransferase | Tryptophan synthesis |
| *Acidovorax* |  | EXV95_09475 | Tryptophan synthase subunit alpha | Tryptophan synthesis |
| *Acidovorax* | *trpD* | EXV95_14185 | Anthranilate phosphoribosyltransferase | Tryptophan synthesis |
| *Acidovorax* | *trpC* | EXV95_14180 | Indole-3-glycerol phosphate synthase TrpC | Tryptophan synthesis |
| *Acidovorax* | *trpB* | EXV95_09470 | Tryptophan synthase subunit beta | Tryptophan synthesis |
| *Acidovorax* |  | EXV95_07155 | Bax inhibitor-1/YccA family protein (PGAP), Anthranilate synthase, amidotransferase component, Para-aminobenzoate synthase, amidotransferase component (RAST) | Tryptophan synthesis |
| *Acidovorax* |  | EXV95_14210 | Anthranilate synthase component I | Tryptophan synthesis |
| *Acidovorax* |  | EXV95_15535 | Shikimate dehydrogenase (PGAP), Phosphoribosylanthranilate isomerase (RAST) | Tryptophan synthesis |
| *Acidovorax* | *xylB* | EXV95_07960 | Xylulokinase | Xylose utilization |
| *Acidovorax* |  | EXV95_23915 | Dihydroxy-acid dehydratase | Xylose utilization |
| *Acidovorax* |  | EXV95_04520 | Phosphomannomutase/phosphoglucomutase | Mannose metabolism |
| *Acidovorax* |  | EXV95_21545 | Mannose-1-phosphate guanylyltransferase/mannose-6-phosphate isomerase | Mannose metabolism |
| *Acidovorax* | *glcF* | EXV95_17865 | Glycolate oxidase subunit GlcF | Glycolate, glyoxylate interconversions |
| *Acidovorax* |  | EXV95_06630 | D-glycerate dehydrogenase (PGAP), Glyoxylate reductase/ Glyoxylate reductase/ Hydroxypyruvate reductase; 2-ketoaldonate reductase, broad specificity (RAST) | Glycolate, glyoxylate interconversions |
| *Acidovorax* | *gph* | EXV95_14225 | Phosphoglycolate phosphatase | Glycolate, glyoxylate interconversions |
| *Acidovorax* |  | EXV95_19255 | FAD-binding protein (PGAP), Glycolate dehydrogenase, subunit GlcD (RAST) | Glycolate, glyoxylate interconversions |
| *Acidovorax* |  | EXV95_17055 | FAD-binding protein (PGAP), D-Lactate dehydrogenase, cytochrome c-dependent (RAST) | Glycolate, glyoxylate interconversions |
| *Acidovorax* |  | EXV95_15340 | SPOR domain-containing protein (PGAP), Glycolate dehydrogenase, FAD-binding subunit GlcE (RAST) | Glycolate, glyoxylate interconversions |
| *Acidovorax* | *ipdC* | EXV95_17420 | Indolepyruvate/phenylpyruvate decarboxylase (PGAP), Pyruvate decarboxylase/alpha-keto decarboxylase (RAST) | Pyruvate Metabolism II |

**S3 Table.** Locus tags for genes of interest in the genome of the reported *Deinococcus* sp. as annotated by RAST/PGAP.

| Organism | Gene | Locus tag | Function/Product | Subsystem |
| --- | --- | --- | --- | --- |
| *Deinococcus* |  | EXW95_01165 | NarK/NasA family nitrate transporter | Nitrate and nitrite ammonification |
| *Deinococcus* | *nirD* | EXW95_01150 | Nitrite reductase small subunit NirD | Nitrate and nitrite ammonification |
| *Deinococcus* | *nirB* | EXW95_01155 | Nitrite reductase large subunit | Nitrate and nitrite ammonification |
| *Deinococcus* | *glnA* | EXW95_15995 | Type I glutamate--ammonia ligase | Ammonia assimilation |
| *Deinococcus* |  | EXW95_16000 | Glutamine synthetase type III | Ammonia assimilation |
| *Deinococcus* |  | EXW95_08825 | Glutamate synthase subunit beta | Ammonia assimilation |
| *Deinococcus* |  | EXW95_10580 | P-II family nitrogen regulator | Ammonia assimilation |
| *Deinococcus* |  | EXW95_10575 | Ammonium transporter | Ammonia assimilation |
| *Deinococcus* |  | EXW95_08830 | Glutamate synthase subunit alpha | Ammonia assimilation |
| *Deinococcus* |  | EXW95_05585 | Thiamine ABC transporter substrate-binding protein | Thiamin biosynthesis |
| *Deinococcus* |  | EXW95_03160 | Hypothetical protein (PGAP), Glycine oxidase ThiO (RAST) | Thiamin biosynthesis |
| *Deinococcus* |  | EXW95_05600 | Thiamine diphosphokinase (PGAP), Thiamin pyrophosphokinase (RAST) | Thiamin biosynthesis |
| *Deinococcus* | *thiE* | EXW95_09825 | Thiamine phosphate synthase (PGAP) Thiamin-phosphate pyrophosphorylase (RAST) | Thiamin biosynthesis |
| *Deinococcus* |  | EXW95_05590 | Iron ABC transporter permease | Thiamin biosynthesis |
| *Deinococcus* | *thiS* | EXW95_09820 | Sulfur carrier protein ThiS | Thiamin biosynthesis |
| *Deinococcus* | *dxs* | EXW95_05435 | 1-deoxy-D-xylulose-5-phosphate synthase | Thiamin biosynthesis |
| *Deinococcus* |  | EXW95_02980 | Tryptophan synthase subunit alpha | Auxin biosynthesis |
| *Deinococcus* | *trpD* | EXW95_06830 | Anthranilate phosphoribosyltransferase | Auxin biosynthesis |
| *Deinococcus* | *trpB* | EXW95_02975 | Tryptophan synthase subunit beta | Auxin biosynthesis |
| *Deinococcus* |  | EXW95_11320 | Phosphoribosylanthranilate isomerase | Auxin biosynthesis |
| *Deinococcus* |  | EXW95_05300 | Heme lyase CcmF/NrfE family subunit | Copper homeostasis |
| *Deinococcus* |  | EXW95_01140 | Copper-translocating P-type ATPase (PGAP), Lead, cadmium, zinc and mercury transporting ATPase (RAST) | Copper homeostasis |
| *Deinococcus* |  | EXW95_15445 | Copper resistance protein CopC | Copper homeostasis |
| *Deinococcus* |  | EXW95_02810 | Cation transporter (PGAP), Cobalt-zinc-cadmium resistance protein (RAST) | Cobalt-zinc-cadmium resistance |
| *Deinococcus* |  | EXW95_15865 | Cation transporter (PGAP), Cobalt-zinc-cadmium resistance protein CzcD (RAST) | Cobalt-zinc-cadmium resistance |
| *Deinococcus* |  | EXW95_00115 | MerR family transcriptional regulator | Cobalt-zinc-cadmium resistance |
| *Deinococcus* |  | EXW95_08335 | Vancomycin resistance protein | Resistance to Vancomycin |
| *Deinococcus* |  | EXW95_09495 | Divalent-cation tolerance protein CutA | Copper homeostasis: copper tolerance |
| *Deinococcus* |  | EXW95_15760 | DNA gyrase subunit B | Resistance to fluoroquinolones |
| *Deinococcus* | *gyrA* | EXW95_16025 | DNA gyrase subunit A | Resistance to fluoroquinolones |
| *Deinococcus* |  | EXW95_16740 | MBL fold metallo-hydrolase | Beta-lactamase |
| *Deinococcus* |  | EXW95_01080 | Transcriptional regulator (PGAP), Cadmium efflux system accessory protein (RAST) | Cadmium resistance |
| *Deinococcus* | *chrA* | EXW95_13065 | Chromate efflux transporter | Resistance to chromium compounds |
| *Deinococcus* |  | EXW95_06835 | Aminodeoxychorismate/anthranilate synthase component II | Tryptophan synthesis |
| *Deinococcus* |  | EXW95_08865 | Aminotransferase class IV (PGAP), Aminodeoxychorismate lyase (RAST) | Tryptophan synthesis |
| *Deinococcus* |  | EXW95_02980 | Tryptophan synthase subunit alpha | Tryptophan synthesis |
| *Deinococcus* |  | EXW95_08860 | Aminodeoxychorismate components I/II (PGAP), Para-aminobenzoate synthase, aminase component/ Para-aminobenzoate synthase, amidotransferase component (RAST) | Tryptophan synthesis |
| *Deinococcus* | *trpD* | EXW95_06830 | Anthranilate phosphoribosyltransferase | Tryptophan synthesis |
| *Deinococcus* | *trpB* | EXW95_02975 | Tryptophan synthase subunit beta | Tryptophan synthesis |
| *Deinococcus* | *trpC* | EXW95_16735 | Indole-3-glycerol phosphate synthase TrpC | Tryptophan synthesis |
| *Deinococcus* |  | EXW95_11320 | Phosphoribosylanthranilate isomerase | Tryptophan synthesis |
| *Deinococcus* | *trpE* | EXW95_06845 | Anthranilate synthase component I | Tryptophan synthesis |
| *Deinococcus* |  | EXW95_09605 | Xylose isomerase | Xylose utilization |
| *Deinococcus* | *xylB* | EXW95_09610 | Xylulokinase | Xylose utilization |
| *Deinococcus* |  | EXW95_11735 | ROK family protein (PGAP), Hexokinase (RAST) | Mannose metabolism |
| *Deinococcus* |  | EXW95_00310 | Class I mannose-6-phosphate isomerase | Mannose metabolism |
| *Deinococcus* |  | EXW95_19205 | Glycoside hydrolase family 2 protein (PGAP), Beta-mannosidase (RAST) | Mannose metabolism |
| *Deinococcus* |  | EXW95_11665 | Alpha-hydroxy-acid oxidizing protein (PGAP), Glycolate oxidase (RAST) | Glycolate, glyoxylate interconversions |
| *Deinococcus* | *glcF* | EXW95_03950 | Glycolate oxidase subunit GlcF | Glycolate, glyoxylate interconversions |
| *Deinococcus* |  | EXW95_03945 | FAD-binding protein (PGAP), Glycolate dehydrogenase subunit GlcD (RAST) | Glycolate, glyoxylate interconversions |
| *Deinococcus* |  | EXW95_03940 | FAD-binding oxidoreductase (PGAP), Glycolate dehydrogenase, FAD-binding subunit GlcE (RAST) | Glycolate, glyoxylate interconversions |
| *Deinococcus* |  | EXW95_07650 | FAD-binding protein (PGAP), D-Lactate dehydrogenase, cytochrome c-dependent (RAST) | Glycolate, glyoxylate interconversions |
| *Deinococcus* |  | EXW95_16175 | Acyl-CoA thioesterase (PGAP), 4-hydroxybenzoyl-CoA thioesterase family active site (RAST) | Ton and Tol transport systems |
| *Deinococcus* |  | EXW95_11815 | Hypothetical protein (PGAP), TonB-dependent receptor (RAST) | Ton and Tol transport systems |
| *Deinococcus* |  | EXW95_13710 | MotA/TolQ/ExbB proton channel family protein | Ton and Tol transport systems |
| *Deinococcus* |  | EXW95_13715 | Biopolymer transporter ExbD | Ton and Tol transport systems |

**S4 Table.** Locus tags for genes of interest in the genome of the reported *Enterobacter* sp. as annotated by RAST/PGAP.

| Organism | Gene | Locus tag | Function/Product | Subsystem |
| --- | --- | --- | --- | --- |
| *Enterobacter* | *norR* | EXW94_22215 | Anaerobic nitric oxide reductase transcription regulator NorR | Nitrosative stress |
| *Enterobacter* | *nsrR* | EXW94_24530 | HTH-type transcriptional repressor NsrR (PGAP), Nitrite-sensitive transcriptional repressor NsrR (RAST) | Nitrosative stress |
| *Enterobacter* |  | EXW94_22220 | Anaerobic nitric oxide reductase flavorubredoxin | Nitrosative stress |
| *Enterobacter* | *norW* | EXW94_22225 | NADH: flavorubredoxin reductase NorW (PGAP), Nitric oxide reductase F\|Rd-NAD(+) reductase (RAST) | Nitrosative stress |
| *Enterobacter* |  | EXW94_04640 | Nitrate reductase subunit alpha | Nitrate and nitrite ammonification |
| *Enterobacter* |  | EXW94_05665 | Nitrate reductase subunit alpha | Nitrate and nitrite ammonification |
| *Enterobacter* |  | EXW94_05715 | ANTAR domain-containing protein (PGAP), Response regulator NasT (RAST) | Nitrate and nitrite ammonification |
| *Enterobacter* |  | EXW94_09235 | MFS transporter (PGAP), Nitrate/nitrite transporter (RAST) | Nitrate and nitrite ammonification |
| *Enterobacter* |  | EXW94_05700 | Nitrate ABC transporter ATP-binding protein | Nitrate and nitrite ammonification |
| *Enterobacter* | *narI* | EXW94_04655 | Respiratory nitrate reductase subunit gamma | Nitrate and nitrite ammonification |
| *Enterobacter* | *narI* | EXW94_05650 | Respiratory nitrate reductase subunit gamma | Nitrate and nitrite ammonification |
| *Enterobacter* | *ntrB* | EXW94_05705 | Nitrate ABC transporter, permease protein | Nitrate and nitrite ammonification |
| *Enterobacter* | *narH* | EXW94_04645 | Nitrate reductase subunit beta | Nitrate and nitrite ammonification |
| *Enterobacter* | *narH* | EXW94_05660 | Nitrate reductase subunit beta | Nitrate and nitrite ammonification |
| *Enterobacter* | *narW* | EXW94_04650 | Nitrate reductase molybdenum cofactor assembly chaperone (PGAP), Respiratory nitrate reductase delta chain (RAST) | Nitrate and nitrite ammonification |
| *Enterobacter* | *narJ* | EXW94_05655 | Nitrate reductase molybdenum cofactor assembly chaperone (PGAP), Respiratory nitrate reductase delta chain (RAST) | Nitrate and nitrite ammonification |
| *Enterobacter* | *nirD* | EXW94_05695 | Nitrite reductase small subunit NirD | Nitrate and nitrite ammonification |
| *Enterobacter* |  | EXW94_09840 | Nitrite reductase large subunit | Nitrate and nitrite ammonification |
| *Enterobacter* | *nirD* | EXW94_09835 | Nitrite reductase small subunit NirD | Nitrate and nitrite ammonification |
| *Enterobacter* | *glnD* | EXW94_19995 | Bifunctional uridylyltransferase/uridylyl-removing protein GlnD | Ammonia assimilation |
| *Enterobacter* |  | EXW94_13295 | Glutamate--ammonia ligase | Ammonia assimilation |
| *Enterobacter* |  | EXW94_10635 | Glutamate synthase small subunit | Ammonia assimilation |
| *Enterobacter* | *glnB* | EXW94_21190 | Nitrogen regulatory protein P-II | Ammonia assimilation |
| *Enterobacter* | *glnE* | EXW94_26820 | Glutamate-ammonia-ligase adenylyltransferase | Ammonia assimilation |
| *Enterobacter* | *amtB* | EXW94_18950 | Ammonium transporter AmtB | Ammonia assimilation |
| *Enterobacter* | *gltB* | EXW94_10640 | Glutamate synthase large subunit | Ammonia assimilation |
| *Enterobacter* |  | EXW94_04640 | Nitrate reductase subunit alpha | Denitrification |
| *Enterobacter* |  | EXW94_05665 | Nitrate reductase subunit alpha | Denitrification |
| *Enterobacter* | *narI* | EXW94_04655 | Respiratory nitrate reductase subunit gamma | Denitrification |
| *Enterobacter* | *narI* | EXW94_05650 | Respiratory nitrate reductase subunit gamma | Denitrification |
| *Enterobacter* | *narW* | EXW94_04650 | Nitrate reductase molybdenum cofactor assembly chaperone (PGAP), Respiratory nitrate reductase delta chain (RAST) | Denitrification |
| *Enterobacter* | *narJ* | EXW94_05655 | Nitrate reductase molybdenum cofactor assembly chaperone (PGAP), Respiratory nitrate reductase delta chain (RAST) | Denitrification |
| *Enterobacter* | *narH* | EXW94_04645 | Nitrate reductase subunit beta | Denitrification |
| *Enterobacter* | *narH* | EXW94_05660 | Nitrate reductase subunit beta | Denitrification |
| *Enterobacter* |  | EXW94_04635 | NarK family nitrate/nitrite MFS transporter | Denitrification |
| *Enterobacter* |  | EXW94_05670 | NarK family nitrate/nitrite MFS transporter | Denitrification |
| *Enterobacter* |  | EXW94_26450 | Autoinducer-2 kinase | Autoinducer 2 (AI-2) transport and processing (lsrACDBFGE operon) |
| *Enterobacter* | *lsrC* | EXW94_26465 | Autoinducer 2 ABC transporter permease LsrC | Autoinducer 2 (AI-2) transport and processing (lsrACDBFGE operon) |
| *Enterobacter* | *lsrA* | EXW94_26460 | Autoinducer 2 ABC transporter ATP-binding protein LsrA | Autoinducer 2 (AI-2) transport and processing (lsrACDBFGE operon) |
| *Enterobacter* | *lsrR* | EXW94_26455 | Transcriptional regulator LsrR | Autoinducer 2 (AI-2) transport and processing (lsrACDBFGE operon) |
| *Enterobacter* |  | EXW94_26480 | Autoinducer 2 (AI-2) aldolase LsrF | Autoinducer 2 (AI-2) transport and processing (lsrACDBFGE operon) |
| *Enterobacter* |  | EXW94_22085 | S-ribosylhomocysteine lyase | Autoinducer 2 (AI-2) transport and processing (lsrACDBFGE operon) |
| *Enterobacter* | *lsrG* | EXW94_26485 | Autoinducer 2 (AI-2) modifying protein LsrG | Autoinducer 2 (AI-2) transport and processing (lsrACDBFGE operon) |
| *Enterobacter* | *lsrB* | EXW94_26475 | Autoinducer 2 ABC transporter substrate-binding protein LsrB | Autoinducer 2 (AI-2) transport and processing (lsrACDBFGE operon) |
| *Enterobacter* | *pgaD* | EXW94_27115 | Biofilm PGA synthesis auxiliary protein PgaD | Biofilm adhesin biosynthesis |
| *Enterobacter* | *pgaA* | EXW94_27130 | Biofilm PGA outer membrane secretin PgaA | Biofilm adhesin biosynthesis |
| *Enterobacter* | *pgaB* | EXW94_27125 | Biofilm PGA synthesis deacetylase PgaB | Biofilm adhesin biosynthesis |
| *Enterobacter* | *pgaC* | EXW94_27120 | Biofilm PGA synthesis N-glycosyltransferase PgaC | Biofilm adhesin biosynthesis |
| *Enterobacter* |  | EXW94_04260 | Response regulator transcription factor CusR (PGAP), Copper-sensing two-component system response regulator CusR (RAST) | Copper homeostasis |
| *Enterobacter* |  | EXW94_06340 | Copper resistance D family protein (PGAP), Copper resistance protein CopD (RAST) | Copper homeostasis |
| *Enterobacter* | *zntA* | EXW94_09430 | Copper-translocating P-type ATPase | Copper homeostasis |
| *Enterobacter* | *copA* | EXW94_19165 | Copper-exporting P-type ATPase CopA | Copper homeostasis |
| *Enterobacter* | *yobA* | EXW94_06345 | CopC domain-containing protein YobA | Copper homeostasis |
| *Enterobacter* | *cueO* | EXW94_22555 | Multicopper oxidase CueO | Copper homeostasis |
| *Enterobacter* |  | EXW94_04265 | Copper sensory histidine kinases CusS | Copper homeostasis |
| *Enterobacter* |  | EXW94_04185 | Response regulator (PGAP), DNA-binding heavy metal response regulator (RAST) | Cobalt-zinc-cadmium resistance |
| *Enterobacter* |  | EXW94_04245 | Cobalt/zinc/cadmium efflux RND transporter, membrane fusion protein, CzcB family | Cobalt-zinc-cadmium resistance |
| *Enterobacter* |  | EXW94_04265 | Copper sensory histidine kinases CusS | Cobalt-zinc-cadmium resistance |
| *Enterobacter* | *dhbA* | EXW94_16195 | 2,3-dihydro-2,3-dihydroxybenzoate dehydrogenase | Siderophore enterobactin |
| *Enterobacter* |  | EXW94_16140 | Enterochelin esterase (PGAP), Enterobactin esterase (RAST) | Siderophore enterobactin |
| *Enterobacter* |  | EXW94_12045 | LPS O-antigen length regulator (PGAP), Ferric enterobactin uptake protein FepE (RAST) | Siderophore enterobactin |
| *Enterobacter* |  | EXW94_16160 | Ferric enterobactin transport system permease protein FepG | Siderophore enterobactin |
| *Enterobacter* |  | EXW94_16175 | Fe2+-enterobactin ABC transporter substrate-binding protein (PGAP), Ferric enterobactin-binding periplasmic protein FepB (RAST) | Siderophore enterobactin |
| *Enterobacter* |  | EXW94_16155 | Ferric enterobactin transport ATP-binding protein FepC | Siderophore enterobactin |
| *Enterobacter* |  | EXW94_16185 | (2,3-dihydroxybenzoyl)adenylate synthase (PGAP), 2,3-dihydroxybenzoate-AMP ligase (RAST) | Siderophore enterobactin |
| *Enterobacter* |  | EXW94_16135 | TonB-dependent siderophore receptor | Siderophore enterobactin |
| *Enterobacter* | *entS* | EXW94_16170 | Enterobactin transporter EntS | Siderophore enterobactin |
| *Enterobacter* | *entF* | EXW94_16150 | Enterobactin non-ribosomal peptide synthetase EntF | Siderophore enterobactin |
| *Enterobacter* | *entC* | EXW94_16180 | Isochorismate synthase EntC | Siderophore enterobactin |
| *Enterobacter* |  | EXW94_16190 | Isochorismatase | Siderophore enterobactin |
| *Enterobacter* | *entH* | EXW94_16200 | Proofreading thioesterase EntH | Siderophore enterobactin |
| *Enterobacter* |  | EXW94_16165 | Fe(3+)-siderophore ABC transporter permease | Siderophore enterobactin |
| *Enterobacter* | *fhuA* | EXW94_20070 | Ferrichrome porin FhuA | Siderophore aerobactin |
| *Enterobacter* | *fhuB* | EXW94_20055 | Fe(3+)-hydroxamate ABC transporter permease FhuB | Siderophore aerobactin |
| *Enterobacter* |  | EXW94_26530 | Siderophore-interacting protein (PGAP), Iron-chelator utilization protein (RAST) | Siderophore aerobactin |
| *Enterobacter* |  | EXW94_07065 | IucA/IucC family siderophore biosynthesis protein | Siderophore aerobactin |
| *Enterobacter* |  | EXW94_07060 | N-acetyltransferase | Siderophore aerobactin |
| *Enterobacter* |  | EXW94_07070 | Lysine 6-monooxygenase | Siderophore aerobactin |
| *Enterobacter* |  | EXW94_07050 | MFS transporter (PGAP), Possible H+-antiporter clustered with aerobactin genes (RAST) | Siderophore aerobactin |
| *Enterobacter* |  | EXW94_07075 | TonB-dependent siderophore receptor | Siderophore aerobactin |
| *Enterobacter* |  | EXW94_07055 | IucA/IucC family siderophore biosynthesis protein (PGAP), N(2)-citryl-N(6)-acetyl-N(6)-hydroxylysine synthase (RAST) | Siderophore aerobactin |
| *Enterobacter* | *fhuC* | EXW94_20065 | Fe3+-hydroxamate ABC transporter ATP-binding protein FhuC | Siderophore aerobactin |
| *Enterobacter* | *fhuD* | EXW94_20060 | Fe(3+)-hydroxamate ABC transporter substrate-binding protein FhuD | Siderophore aerobactin |
| *Enterobacter* | *norR* | EXW94_22215 | Anaerobic nitric oxide reductase transcription regulator NorR | Nitrosative stress |
| *Enterobacter* | *nsrR* | EXW94_24530 | HTH-type transcriptional repressor NsrR (PGAP), Nitrite-sensitive transcriptional repressor NsrR (RAST) | Nitrosative stress |
| *Enterobacter* | *thiQ* | EXW94_22855 | Thiamine ABC transporter ATP-binding protein ThiQ (PGAP), Thiamin ABC transporter (RAST) | Thiamin biosynthesis |
| *Enterobacter* | *thiI* | EXW94_18780 | tRNA 4-thiouridine(8) synthase ThiI | Thiamin biosynthesis |
| *Enterobacter* |  | EXW94_18755 | Thiamine-phosphate kinase | Thiamin biosynthesis |
| *Enterobacter* |  | EXW94_18765 | 1-deoxy-D-xylulose-5-phosphate synthase | Thiamin biosynthesis |
| *Enterobacter* |  | EXW94_22845 | Thiamine ABC transporter substrate binding subunit | Thiamin biosynthesis |
| *Enterobacter* |  | EXW94_01700 | Thiamine kinase | Thiamin biosynthesis |
| *Enterobacter* |  | EXW94_14825 | Thiamine phosphate synthase (PGAP), Thiamin-phosphate pyrophosphorylase (RAST) | Thiamin biosynthesis |
| *Enterobacter* |  | EXW94_07630 | Hydroxyethylthiazole kinase | Thiamin biosynthesis |
| *Enterobacter* | *thiP* | EXW94_22850 | Thiamine/thiamine pyrophosphate ABC transporter permease ThiP | Thiamin biosynthesis |
| *Enterobacter* | *thiS* | EXW94_14815 | Sulfur carrier protein ThiS | Thiamin biosynthesis |
| *Enterobacter* |  | EXW94_05465 | Tryptophan synthase subunit alpha | Auxin biosynthesis |
| *Enterobacter* | *trpD* | EXW94_05450 | Bifunctional anthranilate synthase glutamate amidotransferase component TrpG/anthranilate phosphoribosyltransferase TrpD | Auxin biosynthesis |
| *Enterobacter* | *trpB* | EXW94_05460 | Tryptophan synthase subunit beta | Auxin biosynthesis |
| *Enterobacter* |  | EXW94_03470 | Primary-amine oxidase | Auxin biosynthesis |
| *Enterobacter* | *trpF* | EXW94_05455 | Bifunctional indole-3-glycerol-phosphate synthase TrpC/phosphoribosylanthranilate isomerase TrpF | Auxin biosynthesis |
| *Enterobacter* | *pabA* | EXW94_09865 | Aminodeoxychorismate synthase component 2 | Tryptophan synthesis |
| *Enterobacter* | *trpR* | EXW94_23135 | Trp operon repressor | Tryptophan synthesis |
| *Enterobacter* | *pabB* | EXW94_06175 | Aminodeoxychorismate synthase component 1 | Tryptophan synthesis |
| *Enterobacter* |  | EXW94_05445 | Anthranilate synthase component 1 | Tryptophan synthesis |
| *Enterobacter* |  | EXW94_05455 | Bifunctional indole-3-glycerol-phosphate synthase TrpC/phosphoribosylanthranilate isomerase TrpF | Tryptophan synthesis |
| *Enterobacter* | *trpB* | EXW94_05460 | Tryptophan synthase subunit beta | Tryptophan synthesis |
| *Enterobacter* |  | EXW94_05465 | Tryptophan synthase subunit alpha | Tryptophan synthesis |
| *Enterobacter* |  | EXW94_01650 | Aminodeoxychorismate lyase | Tryptophan synthesis |
| *Enterobacter* |  | EXW94_05450 | Bifunctional anthranilate synthase glutamate amidotransferase component TrpG/anthranilate phosphoribosyltransferase TrpD | Tryptophan synthesis |
| *Enterobacter* | *rcsC* | EXW94_08125 | Two-component system sensor histidine kinase RcsC (PGAP), Xylose ABC transporter, permease protein XylH (RAST) | Xylose utilization |
| *Enterobacter* |  | EXW94_08920 | D-xylose ABC transporter substrate-binding protein | Xylose utilization |
| *Enterobacter* |  | EXW94_08915 | Xylose ABC transporter ATP-binding protein | Xylose utilization |
| *Enterobacter* |  | EXW94_08905 | DNA-binding transcriptional regulator (PGAP), Xylose activator XylR (RAST) | Xylose utilization |
| *Enterobacter* |  | EXW94_08935 | Xylulokinase | Xylose utilization |
| *Enterobacter* | *xylA* | EXW94_08930 | Xylose isomerase | Xylose utilization |
| *Enterobacter* |  | EXW94_02830 | Mannose-6-phosphate isomerase | Mannose metabolism |
| *Enterobacter* |  | EXW94_09815 | Phosphoglycolate phosphatase | Glycolate, glyoxylate interconversions |
| *Enterobacter* | *tolA* | EXW94_16845 | Cell envelope integrity protein TolA | Ton and Tol transport systems |
| *Enterobacter* |  | EXW94_22425 | Protein-L-isoaspartate(D-aspartate) O-methyltransferase | Ton and Tol transport systems |
| *Enterobacter* |  | EXW94_18895 | YbgC/FadM family acyl-CoA thioesterase (PGAP), 4-hydroxybenzoyl-CoA thioesterase family active site (RAST) | Ton and Tol transport systems |
| *Enterobacter* | *tolR* | EXW94_16840 | colicin uptake protein TolR | Ton and Tol transport systems |
| *Enterobacter* |  | EXW94_26530 | Siderophore-interacting protein (PGAP), Iron-chelator utilization protein (RAST) | Ton and Tol transport systems |
| *Enterobacter* |  | EXW94_16135 | TonB-dependent siderophore receptor | Ton and Tol transport systems |
| *Enterobacter* |  | EXW94_07075 | TonB-dependent siderophore receptor | Ton and Tol transport systems |
| *Enterobacter* | *ipdC* | EXW94_20495 | Indolepyruvate decarboxylase (PGAP), Pyruvate decarboxylase/alpha-keto decarboxylase (RAST) | Pyruvate Metabolism II |

**S5 Table.** Locus tags for genes of interest in the genome of the reported *Exiguobacterium* sp. as annotated by RAST/PGAP.

| Organism | Gene | Locus tag | Function/Product | Subsystem |
| --- | --- | --- | --- | --- |
| *Exiguobacterium* |  | EXW93_05545 | Nitrite-sensitive transcriptional repressor NsrR | Rrf2 family transcriptional regulator |
| *Exiguobacterium* |  | EXW93_08865 | Nitrite-sensitive transcriptional repressor NsrR | Rrf2 family transcriptional regulator |
| *Exiguobacterium* |  | EXW93_09085 | Nitrite-sensitive transcriptional repressor NsrR | Rrf2 family transcriptional regulator |
| *Exiguobacterium* | *gltD* | EXW93_09185 | Glutamate synthase subunit beta | Ammonia assimilation, glutamine, glutamate, aspartate, and asparagine biosynthesis |
| *Exiguobacterium* | *gltB* | EXW93_09190 | Glutamate synthase subunit large subunit | Ammonia assimilation, glutamine, glutamate, aspartate, and asparagine biosynthesis |
| *Exiguobacterium* | *glnA* | EXW93_05400 | Type I glutamate--ammonia ligase | Ammonia assimilation, glutamine, glutamate, aspartate, and asparagine biosynthesis, glutamine synthetases |
| *Exiguobacterium* |  | EXW93_03490 | Ammonium transporter | Ammonia assimilation |
| *Exiguobacterium* | *gyrB* | EXW93_16045 | DNA gyrase subunit B | Resistance to fluoroquinolones |
| *Exiguobacterium* |  | EXW93_07255 | GNAT family N-acetyltransferase (PGAP), Streptothricin acetyltransferase, Streptomyces lavendulae type (RAST) | Streptothricin resistance |
| *Exiguobacterium* |  | EXW93_15205 | FAD-dependent oxidoreductase (PGAP), murcuric ion reductase (RAST) | Mercury resistance operon, mercuric reductase |
| *Exiguobacterium* | *cadA* | EXW93_01210 | Cadmium-translocating P-type (PGAP), copper-translocating P-type (RAST) | Copper homeostasis |
| *Exiguobacterium* | *cadA* | EXW93_03090 | Cadmium-translocating P-type (PGAP), copper-translocating P-type (RAST) | Copper homeostasis |
| *Exiguobacterium* |  | EXW93_01080 | Bcr/CflA family efflux MFS (PGAP), Multidrug resistance transporter, Bcr/CflA family (RAST) | Copper homeostasis |
| *Exiguobacterium* |  | EXW93_15425 | Response regulator transcription (RGAP), DNA-binding heavy metal response regulator (RAST) | Cobalt-zinc-cadmium resistance |
| *Exiguobacterium* |  | EXW93_01605 | Cation transporter (PGAP), Cobalt-zinc-cadmium resistance protein (RAST) | Cobalt-zinc-cadmium resistance |
| *Exiguobacterium* |  | EXW93_07385 | Cation transporter (PGAP), Cobalt-zinc-cadmium resistance protein CzcD (RAST) | Cobalt-zinc-cadmium resistance |
| *Exiguobacterium* |  | EXW93_05395 | MerR family transcriptional regulator | Cobalt-zinc-cadmium resistance |
| *Exiguobacterium* |  | EXW93_06885 | MerR family transcriptional regulator | Cobalt-zinc-cadmium resistance |
| *Exiguobacterium* |  | EXW93_07335 | MerR family transcriptional regulator | Cobalt-zinc-cadmium resistance |
| *Exiguobacterium* |  | EXW93_09675 | MerR family transcriptional regulator | Cobalt-zinc-cadmium resistance |
| *Exiguobacterium* | *gyrA* | EXW93_16050 | DNA gyrase subunit A | Resistance to fluoroquinolones |
| *Exiguobacterium* | *parE* | EXW93_08030 | DNA topoisomerase IV subunit B | Resistance to fluoroquinolones |
| *Exiguobacterium* | *parC* | EXW93_08025 | DNA topoisomerase IV subunit A | Resistance to fluoroquinolones |
| *Exiguobacterium* |  | EXW93_05640 | Copper homeostasis protein CutC | Copper homeostasis |
| *Exiguobacterium* | *chrA* | EXW93_13010 | Chromate efflux transporter | Resistance to chromium compounds |
| *Exiguobacterium* |  | EXW93_06400 | Iron ABC transporter substrate-binding protein | Petrobactin-mediated iron uptake system |
| *Exiguobacterium* |  | EXW93_06395 | ABC transporter permease | Petrobactin-mediated iron uptake system |
| *Exiguobacterium* |  | EXW93_06385 | ATP-binding cassette domain-containing protein | Petrobactin-mediated iron uptake system |
| *Exiguobacterium* |  | EXW93_06390 | Iron ABC transporter permease | Petrobactin-mediated iron uptake system |
| *Exiguobacterium* | *thiT* | EXW93_10200 | Energy-coupled thiamine transporter ThiT | Thiamin biosynthesis |
| *Exiguobacterium* |  | EXW93_05895 | FAD-dependent oxidoreductase (PGAP), Glycine oxidase ThiO (RAST) | Thiamin biosynthesis |
| *Exiguobacterium* |  | EXW93_10195 | Thiamine diphosphokinase (PGAP), Thiamin pyrophosphokinase (RAST) | Thiamin biosynthesis |
| *Exiguobacterium* |  | EXW93_08065 | ABC transporter ATP-binding protein (PGAP), Substrate-specific component YkoE of thiamin-regulated ECF transporter for HydroxyMethylPyrimidine (RAST) | Thiamin biosynthesis |
| *Exiguobacterium* |  | EXW93_08075 | Energy-coupling factor transporter transmembrane protein EcfT (PGAP), Transmembrane component YkoC of energizing module of thiamin-regulated ECF transporter for HydroxyMethylPyrimidine (RAST) | Thiamin biosynthesis |
| *Exiguobacterium* | *thiE* | EXW93_02645 | Thiamine phosphate synthase (PGAP), Thiamin-phosphate pyrophosphorylase (RAST) | Thiamin biosynthesis |
| *Exiguobacterium* |  | EXW93_02640 | Hydroxyethylthiazole kinase | Thiamin biosynthesis |
| *Exiguobacterium* | *thiS* | EXW93_05900 | Sulfur carrier protein ThiS | Thiamin biosynthesis |
| *Exiguobacterium* |  | EXW93_04520 | 1-deoxy-D-xylulose-5-phosphate synthase | Thiamin biosynthesis |
| *Exiguobacterium* |  | EXW93_04625 | Tryptophan synthase subunit alpha | Auxin biosynthesis |
| *Exiguobacterium* | *trpD* | EXW93_04605 | Anthranilate phosphoribosyltransferase | Auxin biosynthesis |
| *Exiguobacterium* | *trpB* | EXW93_04620 | Tryptophan synthase subunit beta | Auxin biosynthesis |
| *Exiguobacterium* |  | EXW93_04615 | Phosphoribosylanthranilate isomerase | Auxin biosynthesis |
| *Exiguobacterium* |  | EXW93_00170 | Aminotransferase IV (PGAP), Aminodeoxychorismate lyase (RAST) | Tryptophan synthesis |
| *Exiguobacterium* |  | EXW93_00165 | Aminodeoxychorismate/anthranilate synthase component II | Tryptophan synthesis |
| *Exiguobacterium* |  | EXW93_04615 | Phosphoribosylanthranilate isomerase | Tryptophan synthesis |
| *Exiguobacterium* | *trpE* | EXW93_04595 | Anthranilate synthase component I | Tryptophan synthesis |
| *Exiguobacterium* | *trpC* | EXW93_04610 | Indole-3-glycerol phosphate synthase TrpC | Tryptophan synthesis |
| *Exiguobacterium* | *trpB* | EXW93_04620 | Tryptophan synthase subunit beta | Tryptophan synthesis |
| *Exiguobacterium* | *trpD* | EXW93_04605 | Anthranilate phosphoribosyltransferase | Tryptophan synthesis |
| *Exiguobacterium* |  | EXW93_00160 | Anthranilate synthase component I family protein (PGAP), Para-aminobenzoate synthase, aminase component (RAST) | Tryptophan synthesis |
| *Exiguobacterium* |  | EXW93_04625 | Tryptophan synthase subunit alpha | Tryptophan synthesis |
| *Exiguobacterium* | *manA* | EXW93_00735 | Mannose-6-phosphate isomerase, class I | Mannose metabolism |
| *Exiguobacterium* | *alsS* | EXW93_11860 | Acetolactate synthase AlsS | Alpha-acetolactate operon |

**S6 Table.** Locus tags for genes of interest in the genome of the reported *Paenibacillus* sp. as annotated by RAST/PGAP.

| Organism | Gene | Locus tag | Function/Product | Subsystem |
| --- | --- | --- | --- | --- |
| *Paenibacillus* |  | EXW96_06730 | Alpha/beta hydrolase (PGAP), Trilactone hydrolase (RAST) | Bacillibactin siderophore |
| *Paenibacillus* |  | EXW96_06720 | Iron ABC transporter permease (PGAP), Fe-bacillibactin uptake system FeuB (RAST) | Bacillibactin siderophore |
| *Paenibacillus* |  | EXW96_06715 | Iron-uptake system-binding protein | Bacillibactin siderophore |
| *Paenibacillus* |  | EXW96_06725 | Iron ABC transporter permease | Bacillibactin siderophore |
| *Paenibacillus* |  | EXW96_12940 | Iron ABC transporter permease | Siderophore anthrachelin |
| *Paenibacillus* |  | EXW96_12930 | Siderophore ABC transporter substrate-binding protein | Siderophore anthrachelin |
| *Paenibacillus* |  | EXW96_12935 | ABC transporter ATP-binding protein | Siderophore anthrachelin |
| *Paenibacillus* |  | EXW96_08835 | Rhodanese-like domain-containing protein (PGAP), Sulfur carrier protein adenylyltransferase ThiF (RAST) | Thiamin biosynthesis |
| *Paenibacillus* |  | EXW96_00605 | Transporter substrate-binding domain-containing protein (PGAP), Hydroxymethylpyrimidine ABC transporter, substrate-binding component (RAST) | Thiamin biosynthesis |
| *Paenibacillus* |  | EXW96_05745 | Energy-coupling factor transporter transmembrane protein EcfT | Thiamin biosynthesis |
| *Paenibacillus* | *thiL* | EXW96_22465 | Thiamine-phosphate kinase | Thiamin biosynthesis |
| *Paenibacillus* |  | EXW96_08515 | 1-deoxy-D-xylulose-5-phosphate synthase | Thiamin biosynthesis |
| *Paenibacillus* |  | EXW96_03485 | ABC transporter ATP-binding protein | Thiamin biosynthesis |
| *Paenibacillus* |  | EXW96_05735 | Thiamine ABC transporter permease | Thiamin biosynthesis |
| *Paenibacillus* |  | EXW96_00600 | ABC transporter permease | Thiamin biosynthesis |
| *Paenibacillus* | *thiE* | EXW96_12320 | Thiamine phosphate synthase (PGAP), Thiamin-phosphate pyrophosphorylase (RAST) | Thiamin biosynthesis |
| *Paenibacillus* |  | EXW96_00625 | Hydroxyethylthiazole kinase | Thiamin biosynthesis |
| *Paenibacillus* | *thiS* | EXW96_01645 | Sulfur carrier protein ThiS | Thiamin biosynthesis |
| *Paenibacillus* |  | EXW96_06285 | NarK/NasA family nitrate transporter | Nitrate and nitrite ammonification |
| *Paenibacillus* | *nirD* | EXW96_20380 | Nitrite reductase small subunit NirD | Nitrate and nitrite ammonification |
| *Paenibacillus* |  | EXW96_20375 | NAD(P)/FAD-dependent oxidoreductase (PGAP), Nitrite reductase [NAD(P)H] large subunit (RAST) | Nitrate and nitrite ammonification |
| *Paenibacillus* |  | EXW96_09120 | Tryptophan synthase subunit alpha | Auxin biosynthesis |
| *Paenibacillus* |  | EXW96_06295 | Anthranilate phosphoribosyltransferase | Auxin biosynthesis |
| *Paenibacillus* |  | EXW96_09115 | Tryptophan synthase subunit beta | Auxin biosynthesis |
| *Paenibacillus* |  | EXW96_13925 | Primary-amine oxidase | Auxin biosynthesis |
| *Paenibacillus* |  | EXW96_09110 | Phosphoribosylanthranilate isomerase | Auxin biosynthesis |
| *Paenibacillus* |  | EXW96_25420 | Multicopper oxidase family protein | Copper homeostasis |
| *Paenibacillus* |  | EXW96_18500 | Copper resistance protein CopD | Copper homeostasis |
| *Paenibacillus* |  | EXW96_25350 | Copper chaperone (PGAP), Lead, cadmium, zinc and mercury transporting ATPase (EC 3.6.3.3) (EC 3.6.3.5); Copper-translocating P-type ATPase (EC 3.6.3.4) (RAST) | Copper homeostasis |
| *Paenibacillus* |  | EXW96_24905 | Hypothetical protein (PGAP), Copper resistance protein D (RAST) | Copper homeostasis |
| *Paenibacillus* |  | EXW96_16220 | Hypothetical protein (PGAP), Copper tolerance protein (RAST) | Copper homeostasis |
| *Paenibacillus* |  | EXW96_23300 | Cation transporter (PGAP), Cobalt-zinc-cadmium resistance protein (RAST) | Cobalt-zinc-cadmium resistance |
| *Paenibacillus* |  | EXW96_18530 | Cation transporter (PGAP), Cobalt-zinc-cadmium resistance protein CzcD (RAST) | Cobalt-zinc-cadmium resistance |
| *Paenibacillus* |  | EXW96_24805 | Cytochrome C oxidase subunit II (PGAP), Probable Co/Zn/Cd efflux system membrane fusion protein (RAST) | Cobalt-zinc-cadmium resistance |
| *Paenibacillus* |  | EXW96_23980 | XRE family transcriptional regulator | Cobalt-zinc-cadmium resistance |
| *Paenibacillus* | *cadA* | EXW96_18730 | Cadmium-translocating P-type ATPase | Cobalt-zinc-cadmium resistance |
| *Paenibacillus* |  | EXW96_24475 | Hypothetical protein (PGAP), Vancomycin B-type resistance protein VanW (RAST) | Resistance to Vancomycin |
| *Paenibacillus* |  | EXW96_00575 | Sigma-54-dependent Fis family transcriptional regulator | Zinc resistance |
| *Paenibacillus* |  | EXW96_18345 | Mercury(II) reductase | Mercury reductase/ Mercury resistance operon |
| *Paenibacillus* | *merR* | EXW96_18330 | Hg(II)-responsive transcriptional regulator | Mercury resistance operon |
| *Paenibacillus* | *gyrB* | EXW96_00015 | DNA topoisomerase (ATP-hydrolyzing) subunit B | Resistance to fluoroquinolones |
| *Paenibacillus* | *gyrA* | EXW96_00010 | DNA gyrase subunit A | Resistance to fluoroquinolones |
| *Paenibacillus* | *parE* | EXW96_09360 | DNA topoisomerase IV subunit B | Resistance to fluoroquinolones |
| *Paenibacillus* | *gyrA* | EXW96_09365 | DNA gyrase subunit A (PGAP), Topoisomerase IV subunit A (RAST) | Resistance to fluoroquinolones |
| *Paenibacillus* |  | EXW96_23375 | DEAD/DEAH box helicase (PGAP), Cadmium resistance protein (RAST) | Cadmium resistance |
| *Paenibacillus* |  | EXW96_25465 | ArsR family transcriptional regulator (PGAP), Cadmium efflux system accessory protein (RAST) | Cadmium resistance |
| *Paenibacillus* | *cadA* | EXW96_18730 | Cadmium-translocating P-type ATPase | Cadmium resistance |
| *Paenibacillus* |  | EXW96_20495 | MATE family efflux transporter | Multidrug Resistance Efflux Pumps |
| *Paenibacillus* |  | EXW96_06950 | Efflux RND transporter permease subunit (PGAP), Acriflavin resistance protein (RAST) | Multidrug Resistance Efflux Pumps |
| *Paenibacillus* |  | EXW96_09650 | MATE family efflux transporter | Multidrug Resistance Efflux Pumps |
| *Paenibacillus* |  | EXW96_05285 | MFS transporter | Multidrug Resistance Efflux Pumps |
| *Paenibacillus* |  | EXW96_25155 | Chromate transporter | Resistance to chromium compounds |
| *Paenibacillus* |  | EXW96_20440 | SagB/ThcOx family dehydrogenase (PGAP), TOMM biosynthesis dehydrogenase (protein B) (RAST) | Thiazole-oxazole-modified microcin (TOMM) synthesis |
| *Paenibacillus* |  | EXW96_10815 | TOMM precursor leader peptide-binding protein (PGAP), TOMM biosynthesis cyclodehydratase (protein C) / TOMM biosynthesis docking scaffold (protein D) (RAST) | Thiazole-oxazole-modified microcin (TOMM) synthesis |
| *Paenibacillus* | *pabA* | EXW96_19745 | Aminodeoxychorismate/anthranilate synthase component II | Tryptophan synthesis |
| *Paenibacillus* | *pabC* | EXW96_19750 | 4-amino-4-deoxychorismate lyase | Tryptophan synthesis |
| *Paenibacillus* |  | EXW96_09120 | Tryptophan synthase subunit alpha | Tryptophan synthesis |
| *Paenibacillus* |  | EXW96_19740 | Anthranilate synthase component I family protein (PGAP), Para-aminobenzoate synthase, aminase component (RAST) | Tryptophan synthesis |
| *Paenibacillus* |  | EXW96_09110 | Phosphoribosylanthranilate isomerase | Tryptophan synthesis |
| *Paenibacillus* | *trpE* | EXW96_09095 | Anthranilate synthase component I | Tryptophan synthesis |
| *Paenibacillus* | *trpC* | EXW96_09105 | Indole-3-glycerol phosphate synthase TrpC | Tryptophan synthesis |
| *Paenibacillus* | *trpB* | EXW96_09115 | Tryptophan synthase subunit beta | Tryptophan synthesis |
| *Paenibacillus* |  | EXW96_06295 | Anthranilate phosphoribosyltransferase | Tryptophan synthesis |
| *Paenibacillus* |  | EXW96_23550 | Carbohydrate ABC transporter permease | Xylose utilization |
| *Paenibacillus* |  | EXW96_23205 | Xylose isomerase | Xylose utilization |
| *Paenibacillus* |  | EXW96_21590 | Carbohydrate ABC transporter permease | Xylose utilization |
| *Paenibacillus* | *xylB* | EXW96_14625 | Xylulokinase | Xylose utilization |
| *Paenibacillus* |  | EXW96_07405 | ROK family transcriptional regulator | Xylose utilization |
| *Paenibacillus* |  | EXW96_21790 | NDP-sugar synthase (PGAP), Mannose-1-phosphate guanylyltransferase / Phosphomannomutase (RAST) | Mannose metabolism |
| *Paenibacillus* |  | EXW96_21035 | Mannose-6-phosphate isomerase | Mannose metabolism |
| *Paenibacillus* |  | EXW96_18960 | Cupin domain-containing protein (PGAP), Mannose-1-phosphate guanylyltransferase (GDP) (RAST) | Mannose metabolism |
| *Paenibacillus* |  | EXW96_02920 | D-glycerate dehydrogenase (PGAP), Glyoxylate reductase/ Glyoxylate reductase/ Hydroxypyruvate reductase (RAST) | Glycolate, glyoxylate interconversions |
| *Paenibacillus* |  | EXW96_00990 | (Fe-S)-binding protein | Glycolate, glyoxylate interconversions |
| *Paenibacillus* |  | EXW96_03090 | FAD-binding oxidoreductase | Glycolate, glyoxylate interconversions |
| *Paenibacillus* |  | EXW96_03100 | FAD-binding protein (PGAP), Glycolate dehydrogenase (RAST) | Glycolate, glyoxylate interconversions |
| *Paenibacillus* | *ilvB* | EXW96_20705 | Biosynthetic-type acetolactate synthase (PGAP), Acetolactate synthase, large subunit (RAST) | Acetolactate synthase subunits |

**S7 Table.** Concentrations of indole-3-acetic acid (IAA) produced by the five Lake Erie bacterial isolates after a 24 hour incubation with 5 mM tryptophan in CT-TY media. Colorimetric assay described in the Methods section of the main text.

| Isolate | Concentration of IAA (µM) |
| --- | --- |
| *Exiguobacterium* sp. JMULE1 | 3.3 |
| *Enterobacter* sp. JMULE2 | 39.4 |
| *Deinococcus* sp. JMULE3 | 33.4 |
| *Paenibacillus* sp. JMULE4 | 35.3 |
| *Acidovorax* sp. JMULE5 | 47.3 |


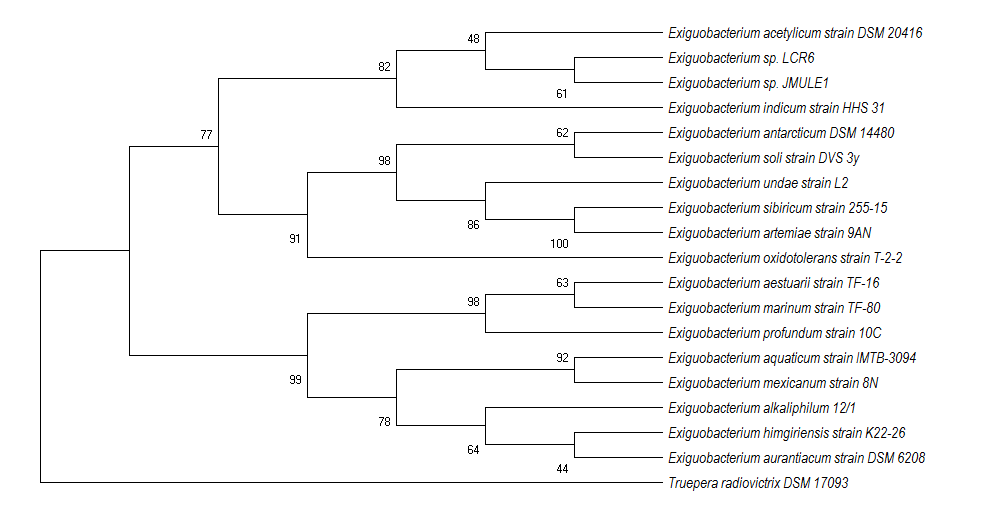


**S1 Fig.** Phylogenetic tree based on the 16S rRNA gene of *Exiguobacterium* sp. JMULE1 and related isolates. Trees were generated in Mega X with the Neighbor-joining method with 1000 for bootstrapping.


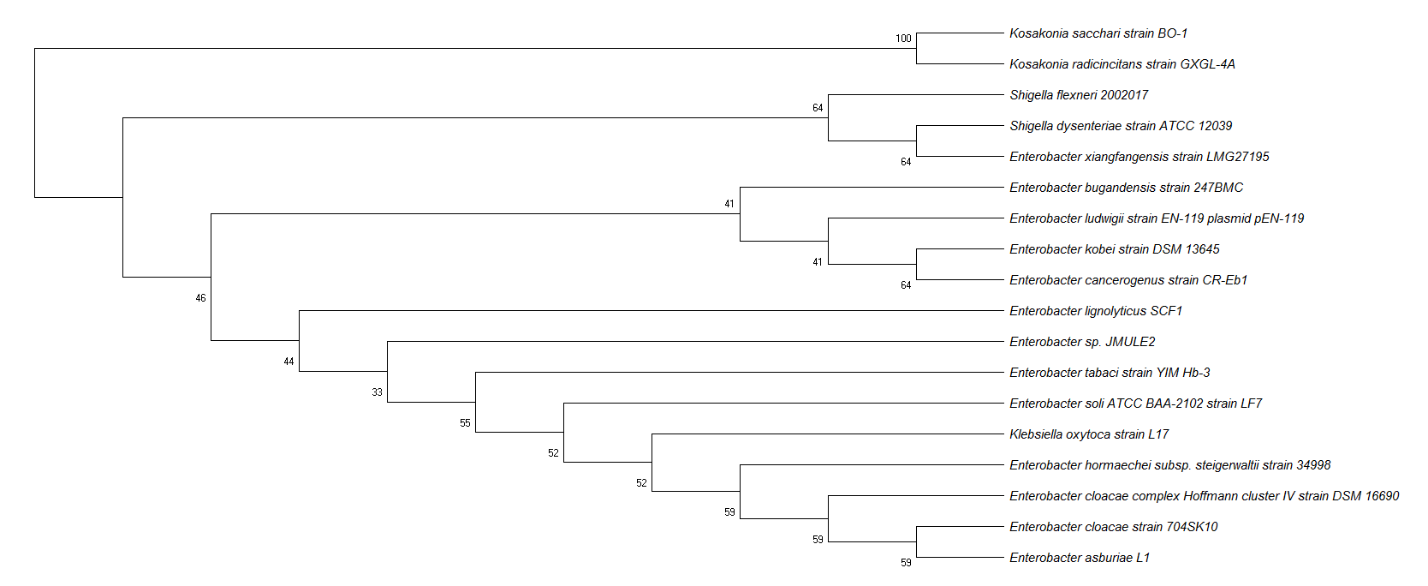


**S2 Fig.** Phylogenetic tree based on the 16S rRNA gene of *Enterobacter* sp. JMULE2 and related isolates. Trees were generated in Mega X with the Neighbor-joining method with 1000 for bootstrapping.


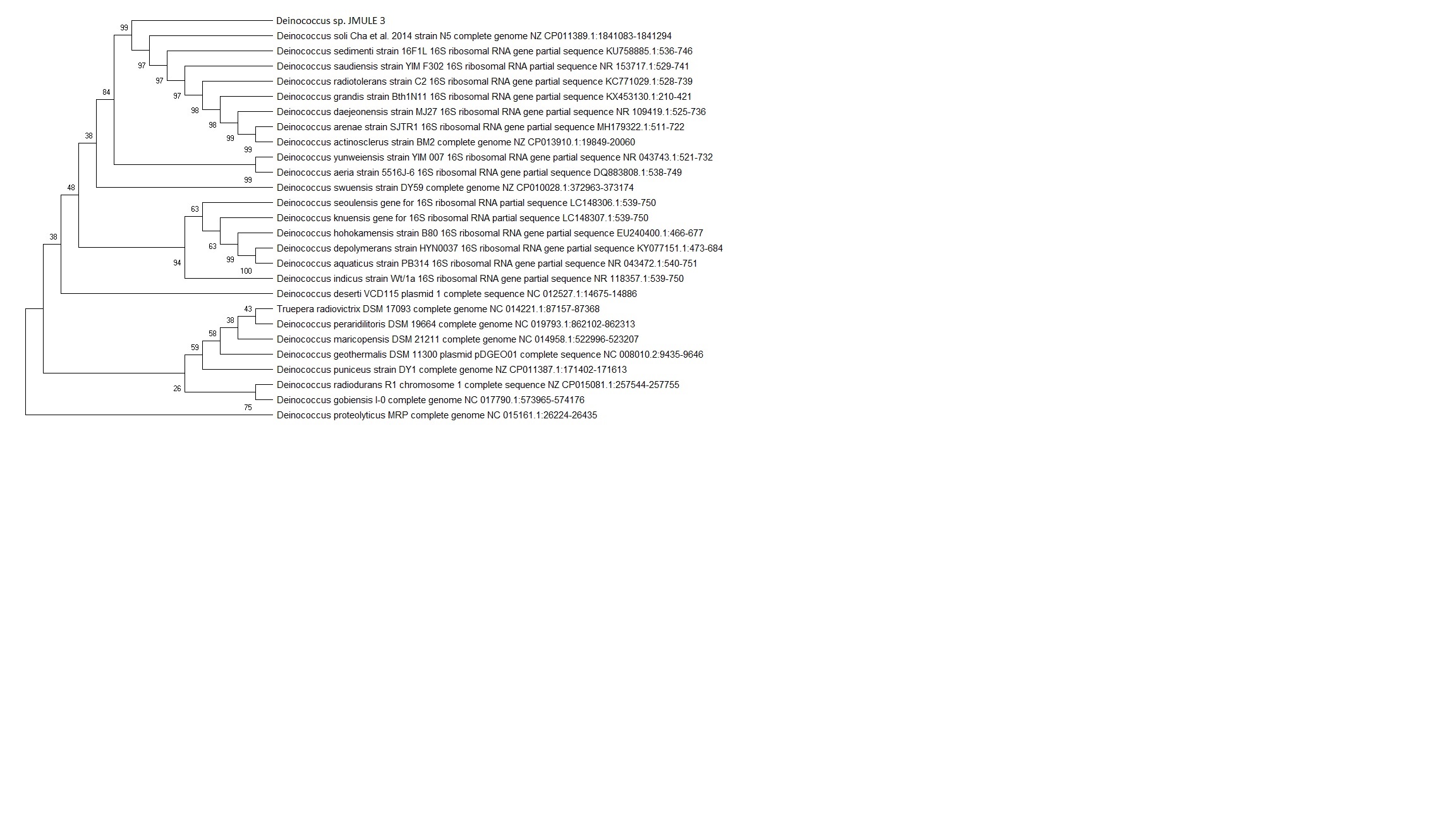


**S3 Fig.** Phylogenetic tree based on the 16S rRNA gene of *Deinococcus* sp. JMULE3 and related isolates. Trees were generated in Mega X with the Neighbor-joining method with 1000 for bootstrapping.


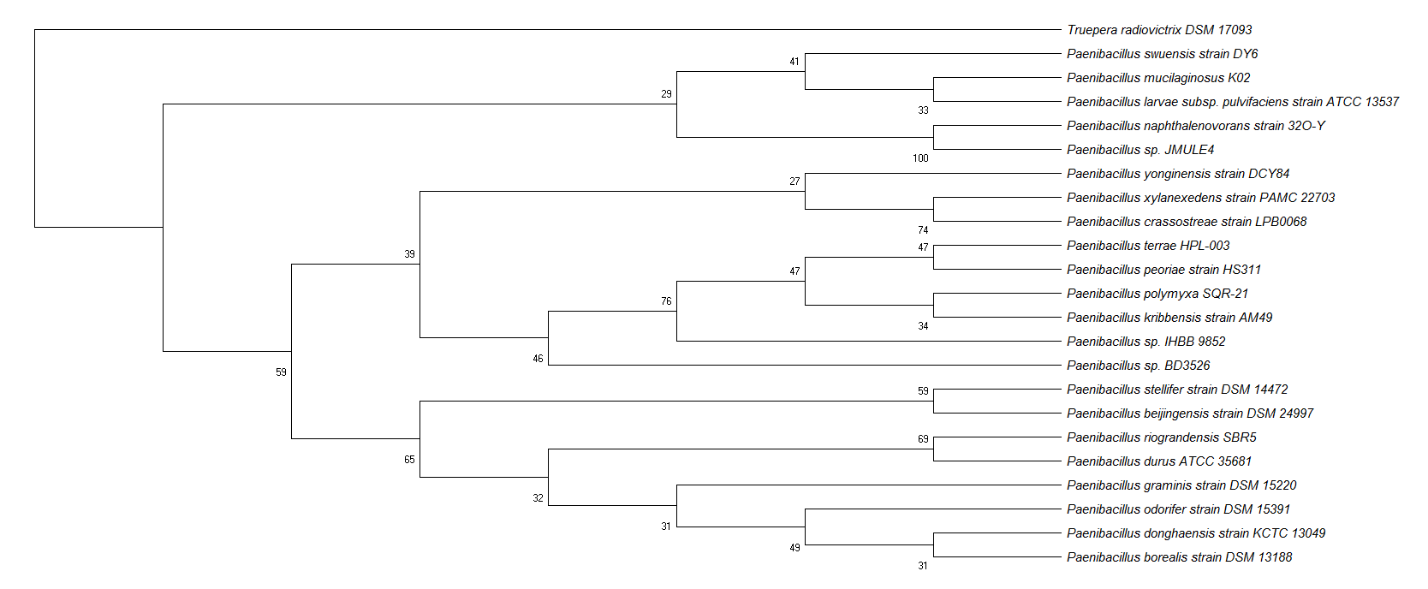


**S4 Fig.** Phylogenetic tree based on the 16S rRNA gene of *Paenibacillus* sp. JMULE4 and related isolates. Trees were generated in Mega X with the Neighbor-joining method with 1000 for bootstrapping.


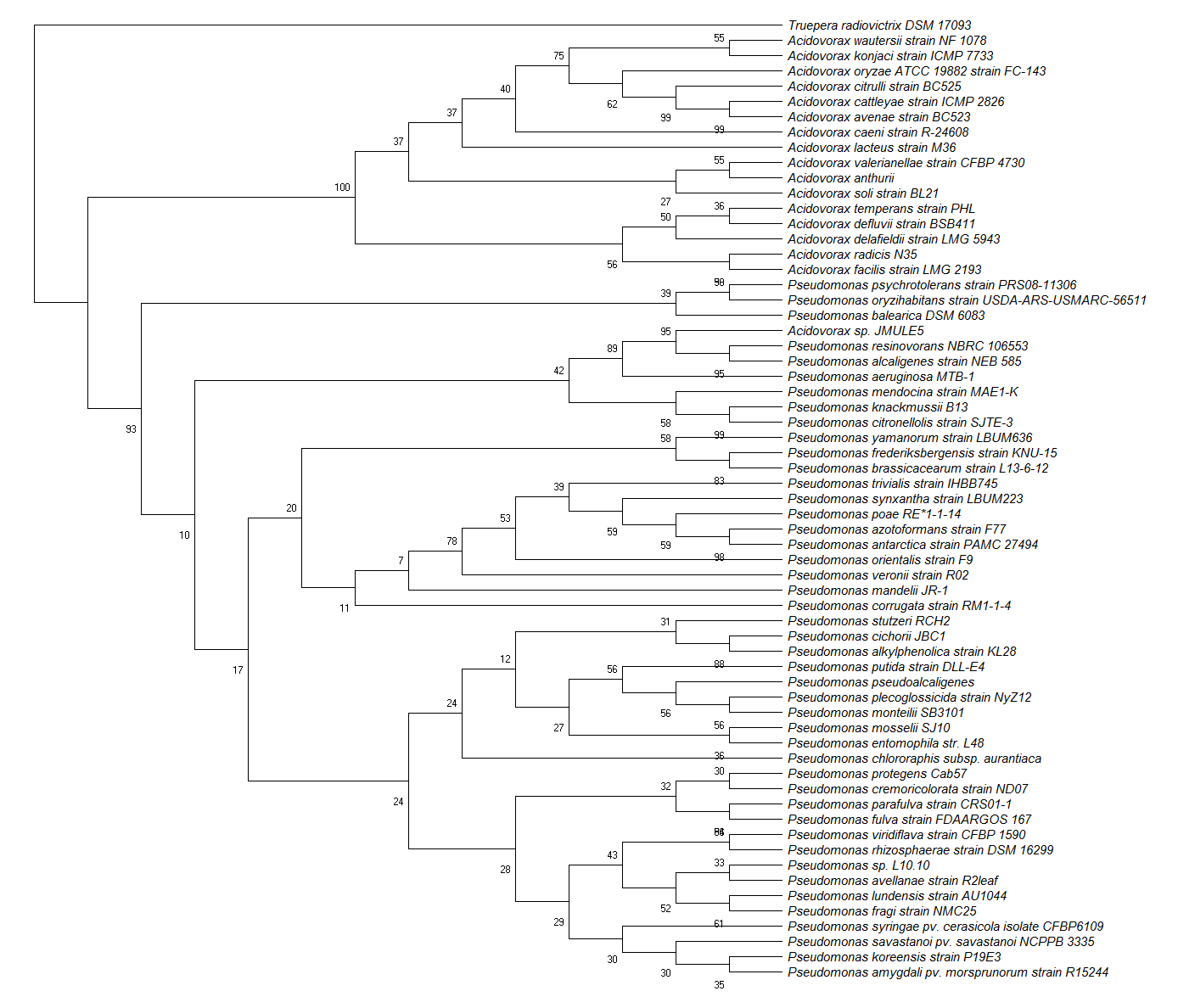


**S5 Fig.** Phylogenetic tree based on the 16S rRNA gene of *Acidovorax* sp. JMULE5 and related isolates. Trees were generated in Mega X with the Neighbor-joining method with 1000 for bootstrapping.
